# Heterosubstituted Derivatives of PtPFPP for O_2_ Sensing and Cell Analysis: Structure–Activity Relationships

**DOI:** 10.1021/acs.bioconjchem.2c00400

**Published:** 2022-10-27

**Authors:** Chiara Zanetti, Rafael Di Lazaro Gaspar, Alexander V. Zhdanov, Nuala M. Maguire, Susan A. Joyce, Stuart G. Collins, Anita R. Maguire, Dmitri B. Papkovsky

**Affiliations:** † School of Biochemistry and Cell Biology, University College Cork, Cork T12 XF62, Ireland; ‡ School of Chemistry, University College Cork, Cork T12 YN60, Ireland; § School of Chemistry and School of Pharmacy, University College Cork, Cork T12 YN60, Ireland

## Abstract

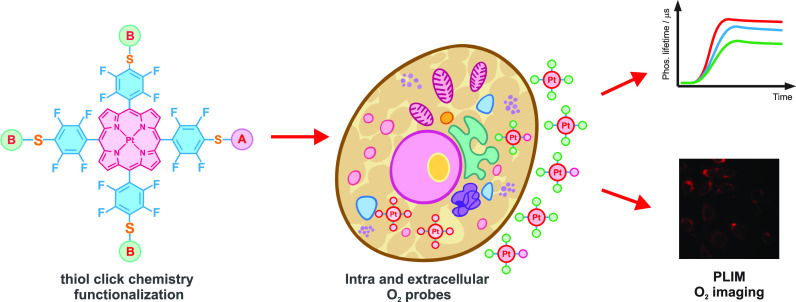

Biological applications of phosphorescent probes for sensing molecular oxygen (O_2_) and bioimaging have gained popularity, but their choice is rather limited. We describe a family of new heterosubstituted phosphorescent bioprobes based on the Pt(II)-tetrakis(pentafluorophenyl)porphyrin (PtPFPP) dye. The probes are produced by simple click modification of its *para*-fluorine atoms with thiols, such as 1/2-thio-glucose, thio-poly(ethylene glycol) (PEG), or cysteamine. The probes were designed to have one cell-targeting moiety and three polar moieties forming a hydrophilic shell. Their chemical synthesis and purification were optimized to produce high reaction yields and easy scale-up. The ability to perform as cell-permeable or -impermeable probes was tuned by the polarity and molecular charge of the bioconjugate. The new PtPFPP derivatives were characterized for their spectral properties and cell-penetrating ability in the experiments with mammalian cell cultures, using a time-resolved fluorescence reader and PLIM imaging detection. Structure–activity relationships were established. Thus, the tri- and tetra-PEGylated structures showed low cell internalization allowing their use as extracellular probes, while cysteamine derivatives performed as efficient intracellular probes. No significant cytotoxicity was observed for all of the probes under the experimental conditions used.

## Introduction

Phosphorescent O_2_-sensing probes facilitate the monitoring of the oxygenation state and O_2_ consumption rates (OCR) of biological samples containing live respiring cells and tissue and link these parameters to vital biochemical processes and cellular responses to stimuli.^1−3^ To date, several types of such O_2_ probes have been developed and applied for the measurement and imaging of O_2_ concentration^4^ and OCR.^5^ Initially, intravascular/intravital O_2_ probes were developed for use in live animals,^6−8^ followed by extracellular probes for in vitro diagnostics and cell-based assays.^9,10^ More recently, intracellular O_2_ probes with cell-penetrating ability have been introduced.^11−15^ Dual pH/O_2_ sensing probes have also been described.^16^ Many of these probes and applications can be used on standard detection platforms.^16^ The key component in all these probes is the phosphorescent indicator moiety that determines their O_2_-sensing and photophysical properties. Pt(II)-tetrakis(pentafluorophenyl) porphyrin (PtPFPP) is an attractive indicator dye for O_2_-sensing assays, as it possesses high brightness and photostability, optimal sensitivity to oxygen, convenient spectral characteristics, availability, and affordable cost. Because of this, PtPFPP is widely used in various polymeric solid-state O_2_ sensors^17^ and nanoparticle-based probes (dispensable aqueous reagents).^11,13^ However, high hydrophobicity and water insolubility prevent its direct use with cells and biological samples as an O_2_ probe. The latter limitation can be overcome by synthesizing more hydrophilic derivatives of PtPFPP and tuning their physical–chemical, O_2_-sensing, and cell-targeting properties. The relatively simple and efficient click modification of PtPFPP via its pentafluorophenyl moieties with thiol- and amine-containing reagents^18−21^ facilitates this work.

Thus, PtPFPP derivatives tetrasubstituted with 1-thio-d-glucose (1Glc) and 1-thio-d-galactose (1Gal) moieties were produced, which possessed hydrophilicity, good solubility in water, and efficient phosphorescent staining of the different types of cells and 3D microtissue models.^22^ Moreover, the very stable S-glycosidic bond in these compounds makes them stable for degradation in biological environments.^23−25^ However, these derivatives showed complex patterns of cell internalization, and they were poorly suited for use as extracellular probes.

In fact, the four saccharide moieties, while increasing cellular uptake and water solubility, improve the amphiphilic properties of porphyrin, facilitating the complex transport of the bioconjugate through the cell membrane.^23,26^ Monosubstituted nitrilotriacetate (NTA) derivatives of PtPFPP were also synthesized, which can chelate heavy metal ions and polypeptide constructs bearing polyhistidine tags.^19^ However, these structures were too hydrophobic with low water solubility and high nonspecific binding to surfaces and biomolecular structures. Conjugates of free porphyrins with saccharide moieties were studied previously, mainly for their photosensitizing activity and possible use in photodynamic therapy^24,27^ or as biomimetics recognized by the cells.^28−30^ Their cell recognition and labeling were studied with a particular focus on drug conjugates with more specific and targeted delivery.^23^ While saccharide moieties improve the water solubility of porphyrin molecules,^24^ their PEGylation was also known to reduce unwanted nonspecific interactions and cellular uptake.^31^ Thus, short PEG fragments (between 400 and 8000 MW) were shown to improve water solubility and bioavailability (serum life), reduce activation of the immune system, and facilitate receptor binding^32,33^ and accumulation of porphyrin sensitizers in tumors.^34^

In this study, we applied the above knowledge to produce new O_2_ probes for both intracellular and extracellular use and study their specificity, recognition, and interaction with cells (via GLUT transporters) and intracellular transport mechanisms. Specifically, we describe a panel of hydrophilic multifunctional phosphorescent oxygen probes produced by click modification of the four pentafluorophenyl moieties in the PtPFPP scaffold with different thiols.^18,20,21^ In particular, we synthesized heterosubstituted bifunctional probes, which contain one cell-targeting monosaccharide moiety (glucose derivatives) and three polar moieties (PEG derivatives or cysteamine) that form a hydrophilic shell. Having synthesized the various heterosubstituted and tetrasubstituted derivatives of PtPFPP, we studied their structure–activity relationships (SAR), particularly the O_2_-sensing characteristics and cell penetration behavior in biological media.

## Results and Discussion

### Rational Design of PtPFPP-Based Phosphorescent Probes

**Figure 1:**
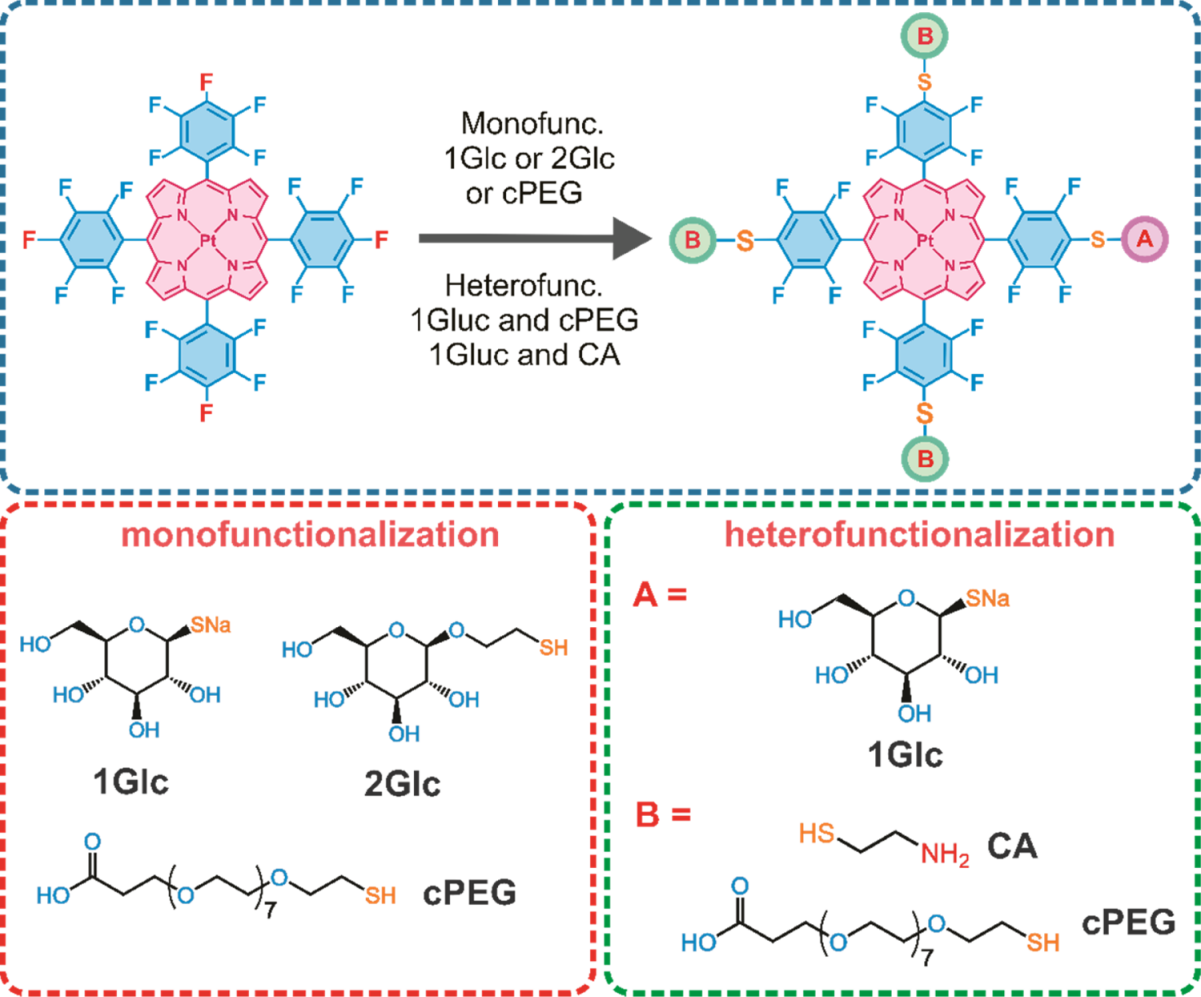
General structures of PtPFPP derivatives produced by click modification with thiols and the schemes of the mono- and hetero-substitution (top panel). The thiols used for the mono- and hetero-functionalization are also shown (bottom panels).

To address the issues with the current probes and better understand their underlying mechanisms, we have decided to synthesize a panel of different hydrophilic PtPFPP derivatives and evaluate them comparatively in aqueous media and biological samples containing live cells. Specifically, we produced a panel of six heterosubstituted derivatives of PtPFPP with glucose, PEG, and cysteamine moieties and studied their biocompatibility and structure–activity relationships. All these compounds contain one biochemical moiety responsible for the interaction with cells or cell surface receptors (e.g., plasma membrane, glucose transporters, GLUTs) and three chemical moieties providing a hydrophilic shell and variable molecular charges (due to carboxy-PEG, cysteamine, methoxy-PEG moieties). Several symmetrical tetrasubstituted derivatives were also synthesized and used for comparison and benchmarking. The general structure and derivatization chemistries of the new bioprobes are shown in Figure 1.

We anticipated that such bifunctional PtPFPP derivatives will show improved hydrophilicity and more predictable and tunable cell internalization behavior, due to their monoglycosylation, variable molecular charge, and surface chemistry. The phosphorescence of these molecular structures can also be altered by substitution, particularly their intensity and lifetime signals in aqueous solutions and biological media. Altogether, this can generate a new family of intracellular or extracellular O_2_-sensing probes for biological applications and also provide more detailed information about their structure–activity relationships.

### Chemical Synthesis of New Compounds and Intermediates

The click modification of the pentafluorophenyl moiety with thiols is known to proceed easily and “cleanly.”^20^^20^ As a consequence, the tetrafunctional PtPFPP is expected to produce five possible products: one mono-, two di- (cis- and trans-), tri-, and tetrasubstituted derivatives. By optimizing the reaction conditions, one can also achieve decent yields of monosubstitution at low molar ratios or almost quantitative yields for tetrasubstituted derivatives at 4–10 M excess of the thiol.

**Figure 2:**
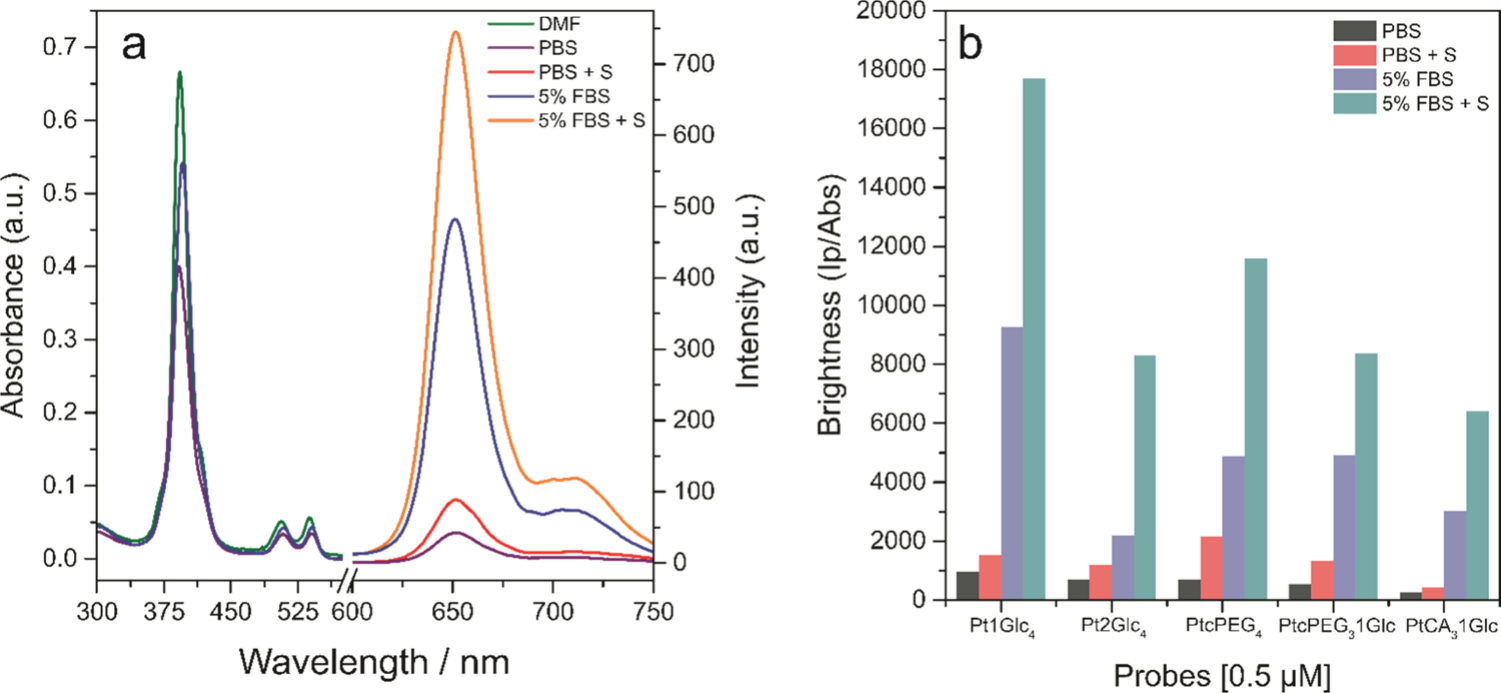
(a) Absorption and emission spectra of PtcPEG_3_1Glc in the different buffers, under air-saturated or deoxygenated conditions, at 3 and 0.5 μM, respectively. (b) Relative brightness of selected PtPFPP derivatives in solution, calculated as sample phosphorescence intensity normalized for its absorbance. PBS, phosphate buffer saline; FBS, fetal bovine serum; and PBS-S, PBS with added 5 mg/mL of KH_2_PO_4_ and 5 mg/mL of Na_2_SO_3_.

Thus, monoglycosylation of PtPFPP with 1Glc or 2Glc thiol at a 1:1 molar ratio in DMF or MeOH containing TEA (see Experimental Procedures) produced key intermediates (compounds 5 and 6; see the Supporting Information) with yields of ∼40%. The first chromatogram in Figure 2 reveals all of the main products in the reaction mixture, with the target 1:1 compound producing the main well-resolved peak, which is easy to separate from the other products. The sequential derivatization of PtPFPP with Glc moieties also shows stepwise increases in hydrophilicity, with sharp, well-resolved, and easily identifiable peaks on RP-HPLC chromatograms (Figure S3). This facilitates synthesis scale-up and purification of target compounds, which in our case was achieved by preparative RP-HPLC. By scaling up the synthesis followed by preparative HPLC purification, compounds 5 and 6 were produced in a pure form, in ∼5 to 10 mg quantities each.

The heterosubstituted derivatives were synthesized by excessive thiolation of monosubstituted intermediates Pt1Glc1 and Pt2Glc1 with anionic hepta- or neutral hexa-poly(ethylene glycols) cPEG-SH and mPEG-SH, respectively. In these cases, almost quantitative yields were achieved (Figure S3). On the other hand, the synthesis of cationic cysteamine (CA) derivatives of PtPFPP required the use of protected Boc-CA (since the CA amino group can also react with the pentafluorophenyl moiety^35^), purification of the target hydrophobic product by RP-HPLC, subsequent deprotection with HCl, and final purification by RP-HPLC (Figure S3). When necessary, TFA salt was subsequently removed by incubating the final product with an equivalent amount of HCl in methanol for 30 min at RT.

Overall, this synthetic work produced six heterosubstituted derivatives: PtcPEG_3_1Glc, PtmPEG_3_1Glc, PtCA_3_1Glc, and their 2Glc counterparts, in 5–10 mg quantities and high yields (70–95% w.r.t. monosubstituted PtPFPP). In addition, three new tetrasubstituted derivatives were also produced: Pt1Glc_4_, Pt2Glc_4_, and PtcPEG_4_. The chemical structure and purity of all these new compounds were confirmed by HPLC, HR-MS, and by ^1^H, ^19^F, and ^13^C NMR spectra (see the Supporting Information). Their main characteristics are summarized in Table 1.

**Table 1:** List of the Newly Synthesized PtPFPP Derivatives and Their Physical Characteristics^a^

conjugate	yield, %	mol charge	MW, g/mol	RT, min
Pt1Glc_4_	96.65	0	1872.47	8.56
Pt2Glc_4_	88.63	0	2048.68	8.88
PtcPEG_4_	95.4	–4	2921.85	13.63
PtcPEG_3_1Glc	97.5	–3	2659.51	12.18
PtmPEG_3_1Glc	65.15	0	2353.24	13.65
PtcPEG_3_2Glc	93.9	–3	2703.56	12.28
PtmPEG_3_2Glc	71.76	0	2397.29	14.72
PtCA_3_1Glc	84.12	+3	1557.34	8.66
PtCA_3_2Glc	35.18	+3	1601.39	8.72

a Notes: retention time (RT) is based on 30 min gradient 0 → 100% of acetonitrile in aqueous 1% TFA and a flow rate of 0.63 mL/min on a YMC-Actus Triart C18, 150 × 4.5 mm^2^ I.D. RP column (YMC).

### Photophysical and O_2_-Sensing Properties

The newly synthesized compounds (Table 1) were subjected to spectroscopic, photophysical, and O_2_-sensing characterization in aqueous media that model the physiological environment. In particular, absorption and emission spectra, phosphorescence lifetimes, and specific brightness (phosphorescent emission normalized to the absorption) were measured for each compound in different buffers without and with protein (5% fetal bovine serum (FBS)) addition, in air-saturated and deoxygenated (addition of 5 mg/mL KH_2_PO_4_, 5 mg/mL Na_2_SO_3_) conditions.

Protein and surfactant additives are known to prevent aggregation of porphyrins in aqueous solutions and influence their self-quenching and quenching by O_2_.^36^

After the initial assessment of solubility, photochemistry, and testing on cultured cells, we focused on four of the eight new structures that were deemed promising for sensing applications. Their characteristics are presented in Table 2 and Figure 2. As described, tri- and tetra-PEGylation of porphyrin compounds, while decreasing hydrophobicity, also increases the tendency of aggregation in aqueous solutions.^36^ In our case, all of the new conjugates in DMF exerted the typical UV–vis absorbance spectra of PtPFPP, with a prominent peak at 393 nm (B band) and two small Q bands in the 500–550 nm region (Figure S4). This small shift of the Soret band is likely due to altered electronic distribution by the conjugation. At the same time, broadened absorption and emission bands were observed in aqueous media, caused by partial aggregation and stacking, which were reduced after the addition of serum.^37,38^

**Table 2:** Photophysical Characteristics of New Derivatives^a^

conjugate	ε_DMF_, M^–1^ cm^–1^	buffer	Abs (λ_max_) [3 μM]	Ip (λ_max_) [0.5 μM]	Ip/Abs [0.5 μM]	LT (μs), 37 °C	QY, Φ
PtPFPP	257 000^b^	PBS + 1% TX-100			1593	60^c^ (CH_2_Cl_2_)	0.088^c^ (CH_2_Cl_2_)
		PBS + 1% TX-100 + sulfite			16 937		
Pt1Glc_4_	227 100^d^	PBS		56 (657)	956	9.1	0.0050
		PBS–sulfite		71 (656)	1528	30.7	0.0079
		PBS + 5% FBS		829 (650)	9261	15.5	0.0481
		PBS + 5% FBS + sulfite		1353 (652)	17 685	37.3	0.0919
Pt2Glc_4_	256 200	PBS	0.32 (404)	22 (658)	691	9.2	0.0036
		PBS–sulfite		26 (657)	1209	20.2	0.0063
		PBS + 5% FBS	0.36 (400)	126 (653)	2184	14.3	0.0113
		PBS + 5% FBS + sulfite		546 (652)	8312	36.7	0.0432
PtcPEG_4_	291 100	PBS	0.28 (392)	35 (653)	705	8.1	0.0037
		PBS–sulfite		90 (653)	2152	25.9	0.0112
		PBS + 5% FBS	0.31 (396)	288 (651)	4878	11.7	0.0253
		PBS + 5% FBS + sulfite		559 (651)	11 599	36.1	0.0603
PtcPEG_3_1Glc	222 400	PBS	0.4 (392)	44 (652)	546	8.2	0.0028
		PBS–sulfite		91 (651)	1320	31.0	0.0069
		PBS + 5% FBS	0.54 (396)	486 (651)	4904	11.7	0.0255
		PBS + 5% FBS + sulfite		747 (650)	8379	34.7	0.0435
PtCA_3_1Glc	230 700	PBS	0.34 (395)	15 (656)	250	11.0	0.0013
		PBS–sulfite		24 (661)	443	34.8	0.0023
		PBS + 5% FBS	0.44 (395)	229 (651)	3025	16.6	0.0157
		PBS + 5% FBS + sulfite		506 (652)	6399	38.6	0.0332

a PBS = phosphate buffer saline. FBS = fetal bovine serum. 10% of sulfite (5 mg/mL KH_2_PO_4_, 5 mg/mL Na_2_SO_3_) was added to deoxygenate the buffer and measure the corresponding lifetime values in air-saturated and deoxygenated conditions. Molar extinction coefficients (ε) were calculated according to the Lambert–Beer Law. Relative quantum yields (Φ) were calculated in relation to PtPFPP. Absorption and emission of the reference dye were measured in deoxygenated aqueous media, assuming its quantum yield as 0.088.

b Reference (39).

c Reference (40).

d Reference (22).

Lifetimes recorded in buffered media at 37 °C were similar for all of the analyzed compounds (Table 2). The particularly lower values were found for PEG derivatives in PBS, while Pt1Glc_4_ and CA derivatives showed longer LT values, reflecting their higher solubility in aqueous media. Upon addition of 5% FBS to the PEGylated derivatives, the brightness increased almost fivefold compared to PBS but still remained considerably lower (∼2 fold) than that for Pt1Glc4 (Figure 2b). The chosen PEG moieties, although not very long, can potentially interact with the porphyrin core and reduce its brightness compared to the symmetrical Pt1Glc_4_, in which the thiol-glucose has less freedom of movement. This can also explain the poor photochemistry of Pt2Glc_4_, in which the 2-carbon linker connects the benzene ring with the rest of the glucose moiety.

### Cellular Uptake and Toxicity of the Probes

**Figure 3:**
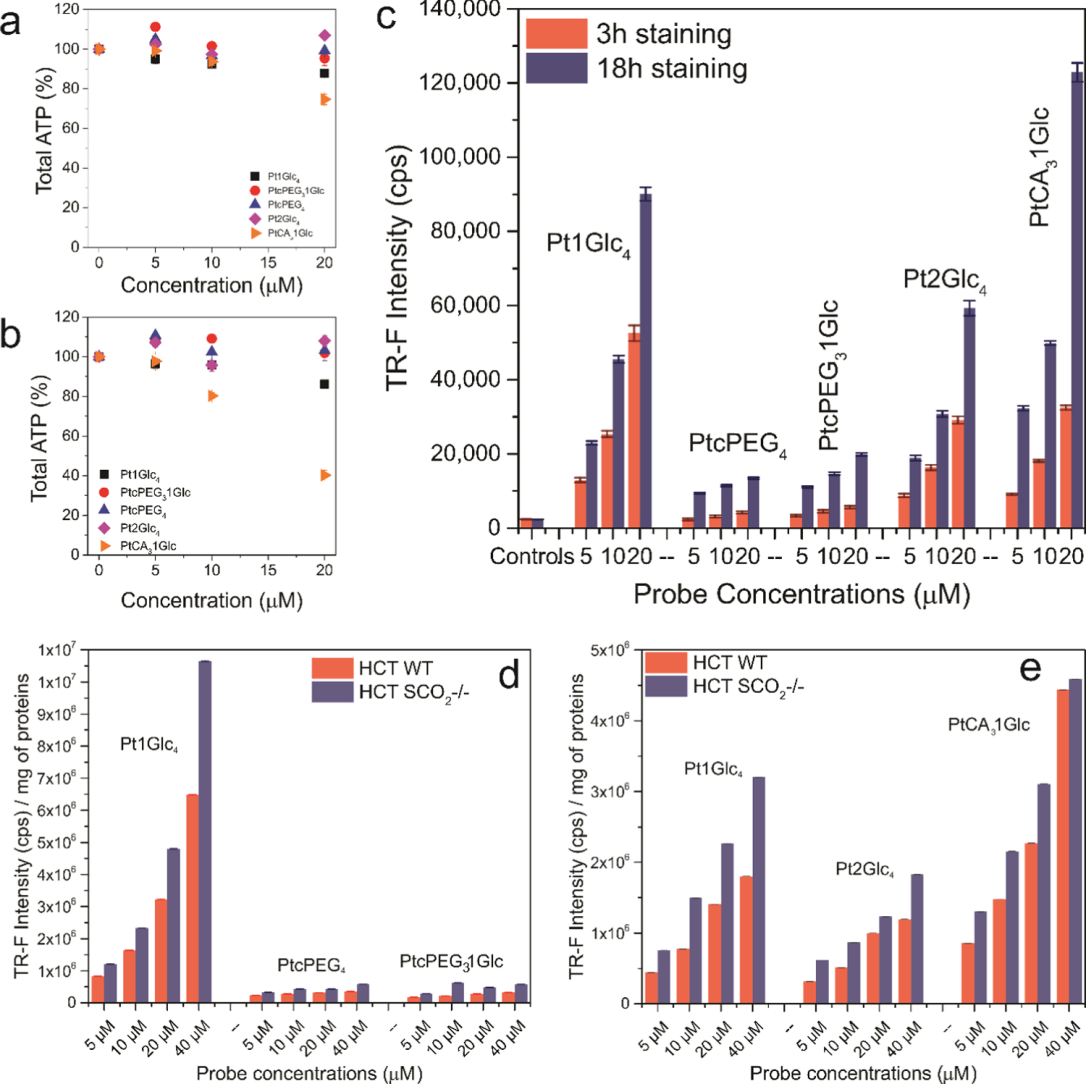
Effects of 3 h (a) and 18 h (b) staining time on the cell viability of MEFs, measured via total ATP content. Comparison of 3 and 18 h cell staining on MEF cells using a range of dye concentrations (c). The intensity of phosphorescence signal normalized for protein content describing the effect of 3 h staining on HCT116 WT (d) and SCO_2_^–/–^ (e) cells. The Pt1Glc_4_ probe was used as a reference.

Cell staining efficiency of the new derivatives was initially analyzed on Murine Embryonic Fibroblasts (MEF) cells, measuring their phosphorescence intensity signals on a Victor 2 reader in TR-F mode. The cells were stained for 3 and 18 h with probe concentrations between 5 and 40 μM (Figure 3). The cellular uptake was significantly lower for three or four PEG conjugates compared to the symmetric Pt1Glc4 and Pt2Glc4. This can be explained by the flexible corona shell and negative charges provided by the multiple carboxy-PEG moieties, which prevent probe interaction with the cell membrane and translocation.^36^ The neutral mPEG derivatives showed behavior similar to carboxy-PEG (data not shown). This is likely due to the ability of PEG chains to adsorb water molecules and increase the effective molecular size of the probe in aqueous solution.^33^

Overall, the amphiphilic nature and lack of cellular receptors/targets for the PEG chains prevent their passive transport through the lipid layer.^23^ On the other hand, the cysteamine derivative showed good cell penetration, similar to or even higher than Pt1Glc_4_. This can be explained by the positive charge of the amino group, which facilitates cell penetration through attractive interactions.^44,54^

The cytotoxicity of each conjugate was also assessed by measuring changes in total ATP in MEF cells. No significant cytotoxicity was seen for all of the conjugates at all of the concentrations and incubation times tested. Only PtCA_3_1Glc showed a small drop in cell viability at concentrations >20 μM. We initially attributed this effect to the residual TFA in the sample,^41−43^ but the use of a specially purified sample gave us the same result. So, we attributed such toxicity to the disruption of the cell membrane mediated by the strong electrostatic attraction between the positively charged probe and the negatively charged lipid bilayer.^44,45^ Interestingly, this cytotoxicity did not correlate with the TR-F signals at these concentrations. The comparison of probes’ cell staining efficiency on the human colorectal carcinoma (HCT116) cell line WT and SCO_2_^–/–^ showed similar results, suggesting GLUTs as one of the main pathways of cellular uptake. SCO_2_^–/–^ is highly glycolytic and nonrespiring human cancer cells, modified by disruption of both alleles of the SCO_2_ gene, which encodes the homonymous protein fundamental for mitochondrial respiration.^46^ Such mutant cells undergo a metabolic switch to glycolysis, which upregulates the expression of glucose transporters.^47^ Particularly, the HCT116 cell line expresses mainly the GLUT1 subtype.^48^

However, other pathways of internalization cannot be ruled out. As previously demonstrated, chelation of extracellular Ca^2+^ by EGTA causes a rapid transient increase in oxygen consumption, which can be monitored by the kinetic measurement of the phosphorescence lifetime of an O_2_-sensitive probe on a TR-F reader.^49^ Calculated lifetime values can then be converted into iO_2_ concentration and plotted over time to evaluate fluctuations in cellular respiration.^50^ At high cell density, changes in local oxygenation can be linked to cellular respiratory activity.

**Figure 4:**
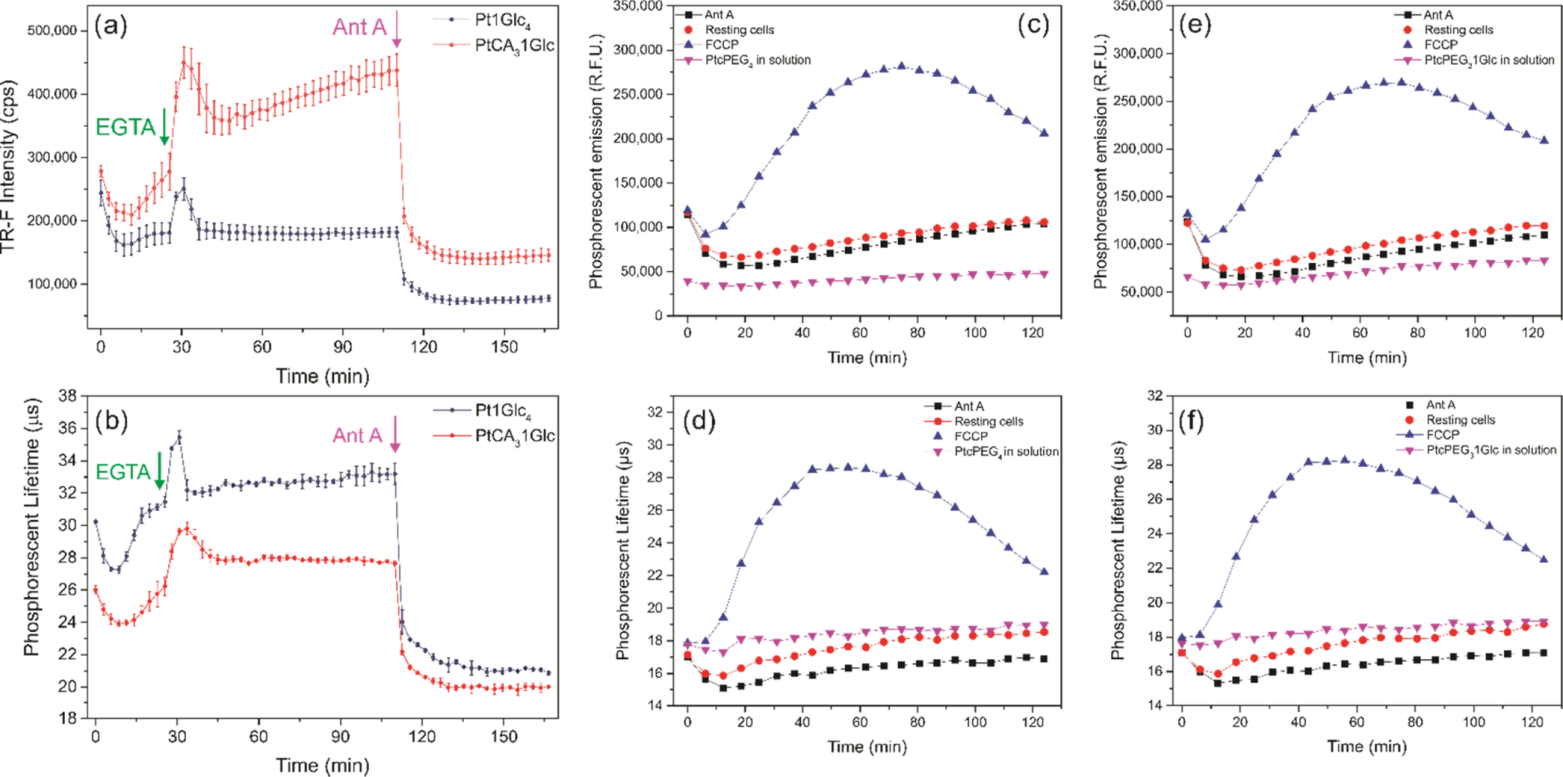
Respiration profiles. TR-F Intensity (a) and lifetime (b) signals produced by the intracellular probes on MEF cells stained at 5 μM for 3 h, measured in the galactose (+) respiration medium. Cellular response to iCa^2+^ depletion with EGTA and inhibition by Ant A on PC12 suspension cells produced by the extracellular probes. Phosphorescent emission and corresponding calculated lifetimes produced by PtcPEG_4_ (c, d, 5 μM) and PtcPEG_3_1Glc (e, f, 5 μM) in the glucose (+) medium, obtaining extracellular respiration profiles upon inhibition of mitochondrial complex III (2 μM Ant A) or FCCP treatments (0.25 μM).

In the absence of full oxygen calibrations for the new probes, only traces of TR-F intensity and LT signals are shown in Figure 4a,b. The graphs include blanks or negative controls (probe alone, no cells), resting cells (positive control), and cells stimulated with metabolic effectors EGTA, antimycin A, and FCCP. One can see that upon cell stimulation with EGTA in the galactose(+) medium,^50^ a marked spike in the intensity and lifetime signal was detected. Inhibition of the response by antimycin A, a potent inhibitor of mitochondrial respiration and cellular O_2_ consumption,^16,50,53^ was also evident. On the other hand, only minimal cellular response was detected in the glucose(+) medium (data not shown). Finally, the analysis of O_2_ gradients was carried out on undifferentiated PC12 cells grown in suspension, to evaluate the usability of the new cell-impermeable derivatives^51^ (Figure 4c–f). We also included the intracellular PtGlc_4_ and the well-established extracellular probe MitoXpress-Xtra as standard references.^9,52^ The brighter probe, Pt1Glc_4_ (see Figure S1), gave a smaller response to FCCP treatment (uncoupler of mitochondrial respiration that increases glycolysis and oxidative phosphorylation rates^16,50,53^) than the tetra- and tri-PEGylated derivatives. The latter probes also showed similar respiration profiles with the MitoXpress-Xtra probe (see Figure S2).

Thus, the new PEGylated derivatives can be used as extracellular probes for the detection of cellular oxygen consumption rates. However, reduced brightness, shorter lifetimes, and the tendency for aggregation make their analytical performance not as good as that of the MitoXpress-Xtra probe.

### Microscopy Analysis of Intracellular Distribution of the O_2_ Probes

**Figure 5:**
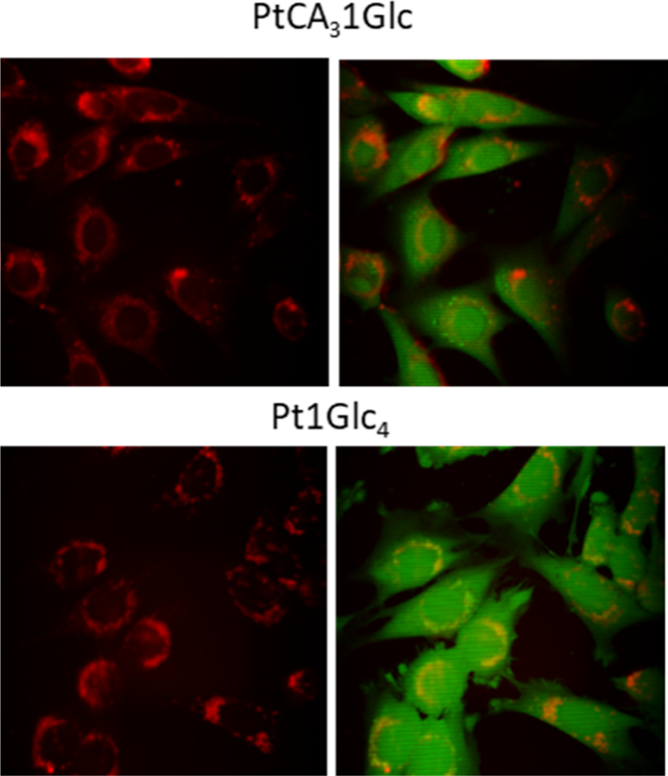
Emission intensity images of the PtCA_3_1Glc (10 μM, 18 h) probe in MEF cells costained with calcein green, measured on a confocal microscope. Pt1Glc_4_ staining localization is also shown for comparison.

To investigate cell staining and intracellular distribution, phosphorescent lifetime imaging experiments on 2D cultures of MEF cells confirmed even and efficient staining for the derivative containing three cysteamine moieties and the absence of any prominent photo- and cytotoxicity. We compared the intracellular staining against Pt1Glc_4_, and as shown in Figure 5, the two probes showed similar perinuclear localization without penetrating the nucleus, as previously described.^22^ However, PEGylated structures did not produce meaningful phosphorescence intensity and PLIM images, confirming that they were not accumulated in cells and were not suitable for intracellular bioimaging applications.

## Conclusions

Using the thiol click modification chemistry, an expanded panel of hydrophilic derivatives of the PtPFPP dye was synthesized and isolated in a pure form in milligram quantities. The new chemical structures, which included three homosubstituted derivatives (Pt1Glc_4_, PtcPEG_4_, and PtmPEG_4_) and six heterosubstituted derivatives (PtcPEG_3_1Glc, PtcPEG_3_2Glc, PtmPEG_3_1Glc, PtmPEG_3_2Glc, PtCA_3_1Glc, and PtCA_3_2Glc) were characterized by spectroscopic techniques and compared to each other.

Subsequently, selected probe structures were evaluated in biological media and the experiments with cells, measuring oxygenation profiles and oxygen consumption rates of the cells and responses to metabolic stimulation. While heterosubstitution with hydrophilic moieties increased the water solubility of PtPFPP, some of the derivatives still exhibited partial aggregation in aqueous media, which is undesirable for biological applications. In particular, modification of the PtPFPP scaffold with PEG oligomers increased the molecular size of the conjugate^33^ and reduced the ability of glucose transporters to internalize such probes. This still allows the use of PEGylated derivatives as extracellular O_2_ probes. Conversely, the positive charge of the cysteamine derivatives improved their cell penetration; however, the selectivity of internalization could be reduced.

Moving forward, evaluation of extracellular application can be further explored, particularly with regard to the symmetric probe PtcPEG4, as well as the testing of alternative heterosubstituted structures to improve cell penetration while maintaining target specificity.

## Experimental Procedures

### Materials

The PtPFPP dye was from Frontier Scientific (Inochem Ltd., Lancashire, U.K.). 1-Thio-β-d-glucopyranoside sodium salt (1Glc), and 2-thioethyl-β-d-glucopyranoside (2Glc) were from Carbosynth Ltd. (Berkshire, U.K.). O-(2-Carboxyethyl)-O′-(2-mercaptoethyl)heptaethylene glycol (cPEG-SH), O-(2-mercaptoethyl)-O′-methyl-hexa(ethylene glycol) (mPEG-SH), 2-(Boc-amino)ethanethiol (Boc-CA) were from Sigma-Aldrich. The cellular ATP assay CellTiter-Glo was from Promega (Madison, WI). The BCA Protein Assay kit was from Thermo Fisher Scientific (Rockford, IL). The MitoXpress-Xtra was from Agilent (Santa Clara, CA). All of the other reagents were from Sigma-Aldrich.

### Synthesis and Purification of PtPFPP Derivatives

Chemical modifications of the PtPFPP scaffold were performed according to the modified methods.^21,22^ Briefly, the corresponding thiol-containing reagent was incubated with PtPFPP in DMF/methanol at molar ratios of 2:1–10:1 in the presence of 10 M excess of the TEA base. Reactions were monitored on an 1100 Series analytical HPLC (Agilent) on a YMC-Actus Triart C18, 150 × 4.5 mm^2^ I.D RP column, using a 30 min gradient 0 → 100% of acetonitrile in aqueous 1% TFA and a flow rate of 0.63 mL/min. Preparative RP-HPLC purification was performed on a Gilson PLC2250, using a YMC-Actus Triart C18, 150 × 20 mm^2^ I.D. RP column (YMC) and the same solvent mixture in a 40 min gradient and a flow rate of 18.9 mL/min.

### Spectral and Photophysical Characterization

UV–vis absorption spectra (range of 350–600 nm) were recorded on an HP8453 diode-array spectrophotometer (Agilent). Phosphorescence spectra (excitation range 300–600 nm and emission range 600–750 nm) and lifetime values were measured on a Cary Eclipse fluorescence spectrometer (Agilent) at 37 °C. NMR spectra were obtained on an AV300 MHz Bruker spectrometer, with chemical shifts relative to residual deuterated CDCl_3_ (ppm).

### High-Resolution Mass Spectrometry (HR-MS)

The analysis of purified PtPFPP derivatives was carried out in a XEVO G2 QToF mass spectrometer (Waters Corporation). Samples were injected by direct infusion after dissolving them in 70:30 ACN/H_2_O (0.3% FA for positive mode, 30 mM TEAA for negative mode). Ionization was performed with a capillary voltage of 2.5 kV and a cone voltage of 40 V in a mass range of 400–3500 *m*/*z*. Source temperature and desolvation temperature were set a 120 and 450 °C, respectively, cone gas flow was set at 50 L/h, and desolvation gas at 800 L/h.

### Cell Culture, Staining, and Toxicity Assessment

Murine embryonic fibroblast (MEF), human colon carcinoma (HCT116) wild-type and SCO_2_^–/–^ mutant cells, and Rat pheochromocytoma (PC12) cells obtained from ATCC (Manassas, VA) were cultured as described before.^11,49,54^ Cell staining experiments were assessed on a TR-F reader Victor 2 (PerkinElmer) at 37 °C, measuring phosphorescence intensity and lifetime signals.

For the staining efficiency experiments, MEFs cells were grown on a 96-well plate for 24 h, seeded at a concentration of 30 000 cells/well, to reach 100% confluence, then incubated with different probe concentrations (5, 10, 20, and 40 μM) for 3 or 18 h, and washed twice and measured in respiration medium containing 10 mM glucose (DMEM, without phenol red and serum free). Phosphorescent intensity signals were recorded at 37 °C on a multilabel plate reader Victor 2 (PerkinElmer) in TR-F mode (340 ± 50 nm excitation, 615 ± 8.5 nm emission filters). Two intensity readings at delay times of 25 and 50 μs were taken, using a gate time of 100 μs and 1 s integration time. Subsequently, measured TR-F intensity signals were converted into lifetime values.^12^ The CellTiter-Glo ATP kit was used to measure probe toxicity on MEF cells via changes in their total ATP.

Cell permeability was also assessed using HCT116 WT and SCO_2_^–/–^. Cells were cultured in McCoy medium supplemented with 10% FBS, 2 mM l-glutamine, and P/S, seeded in a collagen IV-coated 96WP at 20 000 and 30 000 cells/well, grown for 36 h to reach 100% confluence, and then incubated with probes for 3 h as described above. The BCA protein assay was used to evaluate total protein content in cell lysates obtained from HCT WT and SCO_2_^–/–^ seeded on a collagen-coated 6WP at 400 000 and 600 000 cells/well, respectively, and grown for 36 h.

### Respirometry Experiments

Respirometry experiments on intracellular probes were carried out as previously described^49^ on MEF cells seeded at 35 000 cell/well, grown for 30 h to reach high density (>100%), then loaded with intracellular oxygen probes (5 μM), and incubated for 18 h in DMEM containing 10% FBS. EGTA was added at 2.5 mM and antimycin A at 5 μM, with emission intensity measurements taken in gal(+)/glc(−) respiration medium (serum free).

For OCR experiments, PC12 cells were cultured using RPMI 1640 medium supplemented with 5% FBS, 10% horse serum, 10 mM HEPES, and 100 μg/mL penicillin and streptomycin (P/S), pH 7.2. Cells were trypsinized, resuspended in respiration medium (DMEM glc(+), serum free), and counted. Aliquots containing 250 000 cells/well in 100 μL volume mixed with the final dye concentration (Pt1Glc4 at 1 μM, PtcPEG4 and PtcPEG31Glc at 5 μM) were seeded on a 96-well plate in triplicates and treated separately with FCCP (0.25 μM) and Ant A (2.5 μM). Controls without cells and with untreated/nonstained cells were also included and used to correct sensor signals for any drifts unrelated to cellular fluxes. Each well was sealed with 200 μL of mineral oil. Cells suspended in 100 μL of respiration medium containing the MitoXpress-Xtra O_2_ probe were also prepared as the standard reference.

### Bioimaging

MEF cells were seeded on Petri dishes (3.5 cm) at 200 000 in DMEM, grown for 24 h, then loaded with oxygen robes at different concentrations (5, 10, 20, or 40 μM), and incubated for 18 h. The cells were washed three times with fresh medium containing only 1% HEPES and counterstained with calcein green at 1 μM for 30 min. The medium was then replaced with a complete medium, and the cells were imaged on a confocal TCSPC-PLIM microscope^22^ (Becker & Hickl) using an immersion lens adapter at 63× magnification and recorded using SPCImage software (Becker & Hickl). A 488 nm laser was used for the excitation of the calcein green probe, and a 405 nm laser in PLIM mode was used for PtPFPP-based O_2_ probes.

### Data Analysis

All results were obtained from average values produced by at least three replicates, with standard deviations expressed as error bars. To ensure consistency, all of the experiments were performed in duplicate or triplicate.

## Supporting Information Available

More detailed information is provided on the chemical synthesis of the new chemical structures; their NMR and QToF mass spectra and HPLC traces; and additional figures on cell respirometry (PDF)

## Abbreviations

PtPFPP: Pt(II)-tetrakis(pentafluorophenyl)porphyrin
Glc: glucose
GLUT: glucose transporter
TFA: trifluoroacetic acid
FBS: fetal bovine serum
PBS: phosphate buffer saline
RP-HPLC: reversed-phase high-pressure liquid chromatography
