# Supplementary material for: Heterosubstituted Derivatives of PtPFPP for O2 Sensing and Cell Analysis: Structure–Activity Relationships
Source: Bioconjug Chem. 2022 Oct 27;33(11):2161–9. doi: 10.1021/acs.bioconjchem.2c00400 (PMC9673148; doi:10.1021/acs.bioconjchem.2c00400)
Supplement: Supplementary file 1 — bc2c00400_si_001.pdf [file bc2c00400_si_001.pdf]

Supporting Information

Hetero-substituted derivatives of PtPFPP for O_2 sensing and cell analysis: structure-activity relationships

Chiara Zanetti[1], Rafael Di Lazaro Gaspar[1], Alexander V. Zhdanov[1], Nuala M. Maguire[2], Susan A. Joyce[1], Stuart G. Collins[2], Anita R. Maguire[3], Dmitri B. Papkovsky[1, *]

1) School of Biochemistry and Cell Biology, University College Cork, Cork, T12 XF62, Ireland

2) School of Chemistry, University College Cork, Cork, T12 YN60, Ireland

3) School of Chemistry and School of Pharmacy, University College Cork, Cork, T12 YN60, Ireland

* - Corresponding author. Email: d.papkovsky@ucc.ie

Table of Contents

1. Chemical synthesis S2
2. NMR spectra and chemical structures S7
3. Q-TOF Mass spectra S24
4. Supplementary figures S29

1. Chemical synthesis

1.1. *Pt1Glc$_4$ (**1**) synthesis:* PtTFPP (0.585 mg, 500 nmoles) was dissolved in 1.05 ml of DMF, mixed with thioglucose (1.09 mg, 5 µmoles) in 0.45 ml of MeOH containing TEA (1.63 µl, 10 µmoles), incubated for 5h at 40°C, followed by RP-HPLC purification (0.905 mg 96.65%). Purity (HPLC): 93.3%. ^{1}H NMR ((CD_3)$_2$CO, 600 MHz, (C*H*$_3$)$_2$CO=2.07 ppm) δ ppm= 3.48-4.51 (4H, m, 4’-Glc*H*), 3.55-3.63 (12H, m, 2’3’5’-Glc*H*), 3.77 (4H, dd, J1,2=11.66, 6.51, 6’-Glc*H*), 4.03 (4H, dd, J1,2= 11.87, 2.42, 6’b-Gluc*H*), 5.23 (4H, d, J = 8.78 Hz, 1’-Glc*H*), 9.24, (8H, s, β-pyrrole*H*). 13C NMR ((CD_3)$_2$CO, 600 MHz, (CD_3)$_2$CO=28.96, 205.45 ppm) δ ppm= 62.3 (6’-Glc*C*), 70.9 (4’-Glc*C*), 75.06 (2’-Glc-*C*), 78.8 (3’-Glc*C*), 81.53 (5’-Glc*C*), 84.82 (1’-Glc*C*), 107.35 (meso*C*), 113.99 (1-Ph*C*), 119.4 (4-Ph*C*), 131.9 (β-pyrrole*C*), 141.28 (α-pyrrole*C*), 145.26-148.4 (3,5-Ph*C* and 2,6-Ph*C*). 19F NMR (CD_3OD, 300 MHz) δ ppm= -134.86 (8F, dd, $J^{1,2}$= 24.89, 11.73, *meta*- 3,5-Ph*F*Glc), -140.52 (8F, dd, $J^{1,2}$=24.61, 11.73, *ortho*- 2,6-Ph*F*Glc). UV–vis (DMF, path length = 1 cm, 25°C): λ(nm)=393, 538, 506. PL (c = 0.2µM, DMF, path length = 1.0 cm, λ_{ex} = 390 nm, 25 °C): λ(nm) = 650, 707.

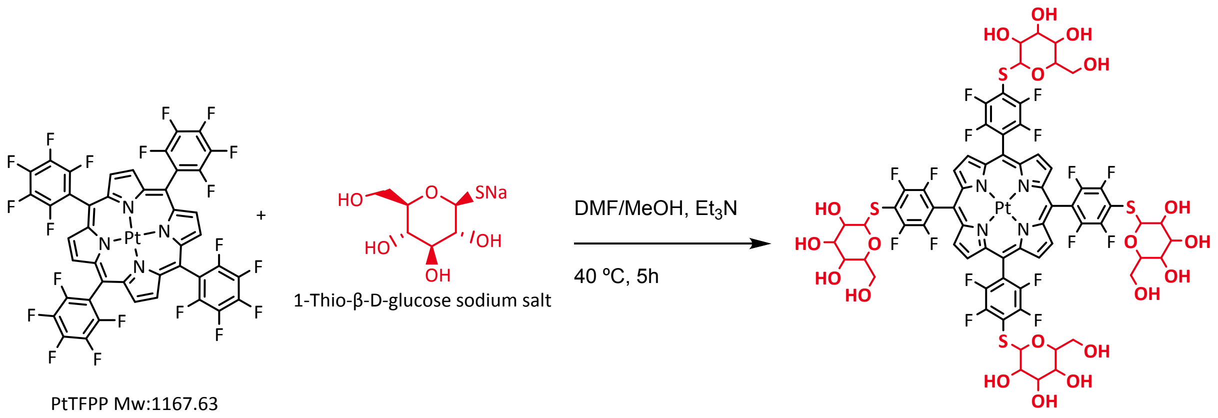

(1) - $C_{68}H_{52}F_{16}N_4O_{20}PtS_4$
Mw: 1872.47

1.2. *Pt2Glc$_4$ (**2**)*. PtTFPP (0.585 mg, 500 nmoles) dissolved in 0.46 ml of DMF was mixed with thioglucose (1.2 mg, 5 µmoles) in 0.24 ml of MeOH containing TEA (1.63 µl, 10 µmoles), incubated overnight at 40°C, then purified by RP-HPLC (0.91 mg, yield 88.63%). Purity (HPLC): 96.8%. QToF high resolution mass spectrometry: m/z for $C_{76}H_{66}F_{16}N_4O_{24}PtS_4^{2-}$ ($[M-2H]^{2-}$) cacld 1023.11354, found 1023.1216. ^{1}H NMR (CD_3OD, 300 MHz, C*H*D$_2$OD = 3.33 ppm) δ ppm= 3.28-3.5 (16H, m, 4’,2’,3’,5’-Glc*H*), 3.54 (2H, t, J=6.4, SC*H*$_2$CH$_2$-Glc), 3.75 (4H, dd, $J^{1,2}$=11.81, 5.25

Hz, 6'-Glc*H*),3.94-3.98 (4H, m, 6'-Glc*H*), 4.08 (4H, dt, J =17.1, 6.42 Hz, SCH_2CH_2-Glc), 4.33 (4H, dt, J = 16.9, 6.42 Hz, SCH_2CH_2-Glc), 4.47 (4H, d, J=7.73 Hz, 1'-Glc*H*), , 9.1 (8H, s, β-pyrrole*H*). ^{13}C NMR (CD_3OD, 600 MHz, CD_3OD=49.0 ppm) δ ppm= 49.85 (S-CH_2), 62.89 (6'-Glc*C*), 70.69 (S-CH_2CH_2), 71.71 (4'-Glc-*C*), 75.24 (2'-Glc-*C*), 78.11 (3'-Glc*C*), 78.14 (5'-Glc*C*), 104.63 (1'-Glc*C*), 108.51 (meso*C*), 117.92 (1-Ph*C*), 120.6 (4-Ph*C*), 132.73 (β-pyrrole*C*), 142.66 (α-pyrrole*C*), 146.73-149.4 (3,5-Ph*C* and 2,6-Ph*C*). ^{19}F NMR (CD_3OD, 300 MHz) δ ppm= -135.71 (8F, dd, J1,2= 24.32, 11.52, *meta*- 3,5-Ph*F*Glc), -140.32 (8F, dd, J1,2=24.61, 11.73, *ortho*- 2,6-Ph*F*Glc). UV–vis (DMF, path length = 1.0 cm, 25°C): λ(nm)=393, 538, 506. PL (c = 0.2μM, DMF, path length = 1 cm, λex = 390 nm, 25 °C): λ(nm) =651, 705.

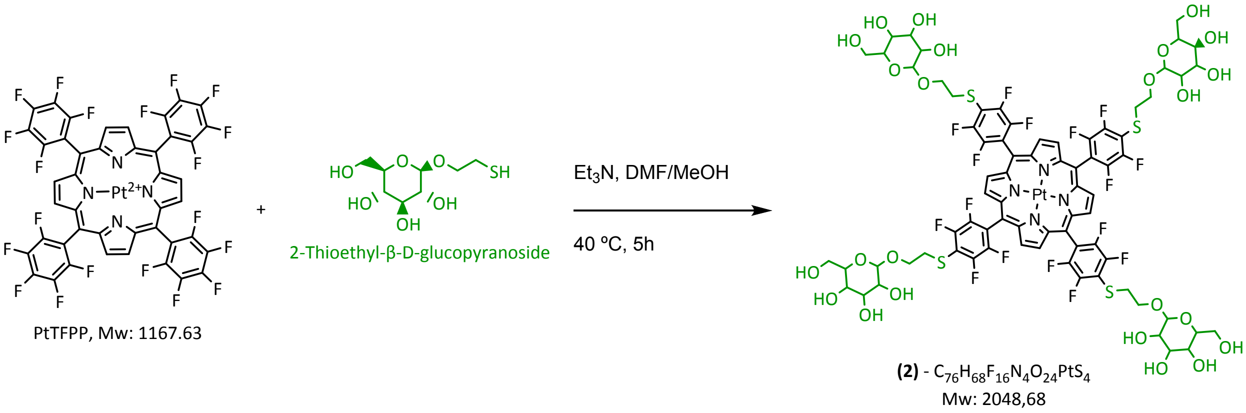

1.3. *PtcPEG$_4$ (**3**),* PtTFPP (0.585 mg, 500 nmoles) dissolved in 1.5 ml of DMF was mixed with cPEG7-SH (2.3 mg, 5 μmoles), or mPEG7-SH (1.78 mg, 5 μmoles), in presence of TEA (1.63 μl, 10 μmoles), and incubated 5h at 40°C, then purified by RP-HPLC (1.39mg, yield 95.4%). Purity (HPLC): 94.9%. QToF high resolution mass spectrometry: m/z for $C_{120}H_{154}F_{16}N_4O_{40}PtS_4^{2-}$ ([M-2H]$^{2-}$) cacld 1459.4229, found 1459.1919. ^{1}H NMR (600 MHz, ($CDCl_3$, $CHCl_3$= 7.2 ppm) δ ppm= 2.5 (8H, t, J=6.12 Hz , CH_2-COOH), 3.38 (8H, t, J=5.96 Hz, S-CH_2), 3.5-3.7 (120H, m, O-$(CH_2)_{60}$), 3.87 (8H, t, J=5.95, SCH_2-CH_2), 8.79 (8H, s, β-pyrrole*H*). ^{13}C NMR (600 MHz, $CDCl_3$, $CHCl_3$ =77.04) δ ppm= 34.42 (S-CH_2), 34.98 (COOH-CH_2), 66.52 (COOH-CH_2CH_2), 70.21 (S-CH_2 CH_2), 70.21-70.74 (PEG-*C* chain), 107.15 (meso*C*), 116.82 (1-Ph*C*), 129.3 (4-Ph*C*), 131.15 (β-pyrrole*C*), 141 (α-pyrrole*C*), 145.14-147.59 (3,5-Ph*C* and 2,6-Ph*C*), 174.33 (-*C*=OOH). ^{19}F NMR (300 MHz, $CDCl_3$) δ ppm= -133.54 (8F, dd, J1,2= 25.22, 12.03, *meta*- 3,5-Ph*F*Glc), -137.06 (8F, dd, $J^{1,2}$=24.65, 11.75, *ortho*- 2,6-Ph*F*Glc). UV–vis (DMF, path length = 1.0 cm, 25°C): λ(nm)=393, 538, 506. PL (c = 0.2μM, DMF, path length = 1.0 cm, λ_{ex} = 390 nm, 25 °C): λ(nm) =651, 701.

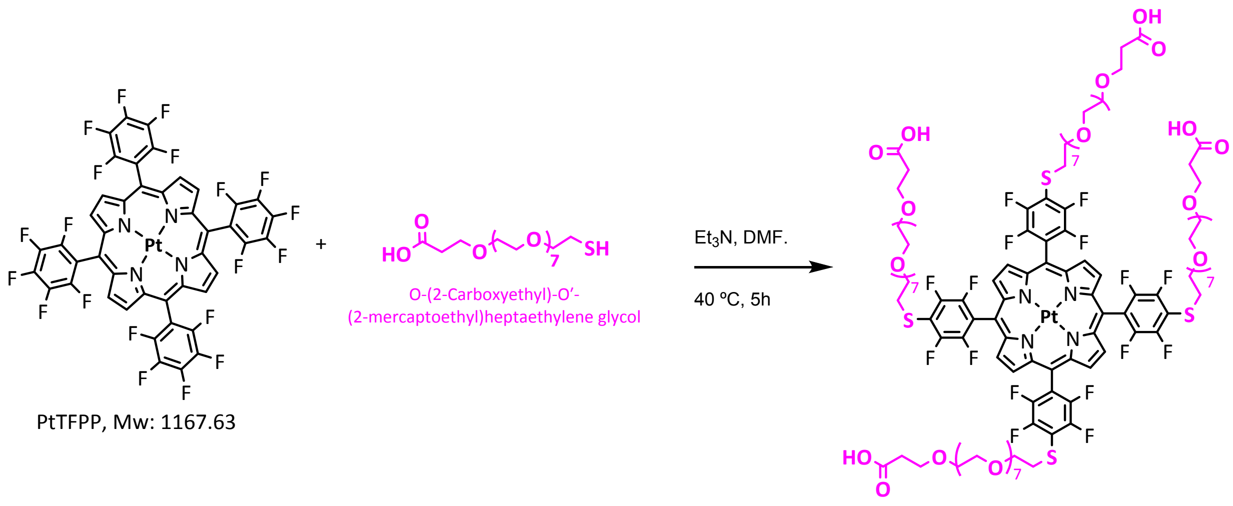

PtTFPP, Mw: 1167.63

(3) - $C_{120}H_{156}F_{16}N_4O_{40}PtS_4$
Mw: 2921.85

*PtmPEG₄ (**4**)* was synthesized likewise using mPEG-SH (1.78 mg, 5 µmoles) obtaining 1.03 mg, yield 81.86%. Purity (HPLC): 98.6%.

1.4. *Pt1Glc₁ (**5**).* PtTFPP (0.585 mg, 500 nmoles) dissolved in 1.2 ml of DMF, with 1-Thio-β-D-glucose sodium salt (0.218 mg, 1 µmole) dissolved in 0.3 ml of MeOH containing TEA (1.63 µl, 10 µmoles) for 5h at 40°C, monitored by analytical RP-HPLC (0.27 mg, yield 39.8%). Purity (HPLC): 99.6%. QToF high resolution mass spectrometry: m/z for $C_{52}H_{24}F_{19}N_5O_6PtS^-$ ($[M+CH_3CN+H_2O]^-$) cacld 1402.07994, found 1402.0421. ^{1}H NMR (300 MHz, $CDCl_3$,$CHCl_3$=7.28ppm)) δ ppm= 3.75-4.17 (6H, m, 2',3',4',5',6'a,6'b-Gluc*H*), 5.2 (1H, d, J = 8.8 Hz, 1'-Glc*H*), 8.73-8.82 (8H, m, β-pyrrole*H*). ^{19}F NMR (300 MHz, $CDCl_3$) δ ppm= -132.00 (2F, dd, $J^{1,2}$= 25.68, 12.53, *meta*- 3,5-Ph*F*Glc), -135.72 (2F, dd, $J^{1,2}$=24.82, 11.92, *ortho*- 2,6-Ph*F*Glc), -136.42,-136.65 (6F, m, *meta*- 3,5-Ph*F*), -151.05,-151.37 (3F, m, *para*- 4-Ph*F*), -161.1,-161.4 (6F, m, *ortho*- 2,6-Ph*F*). UV–vis (DMF, path length = 1.0 cm, 25°C): λ(nm)=390, 538, 505.

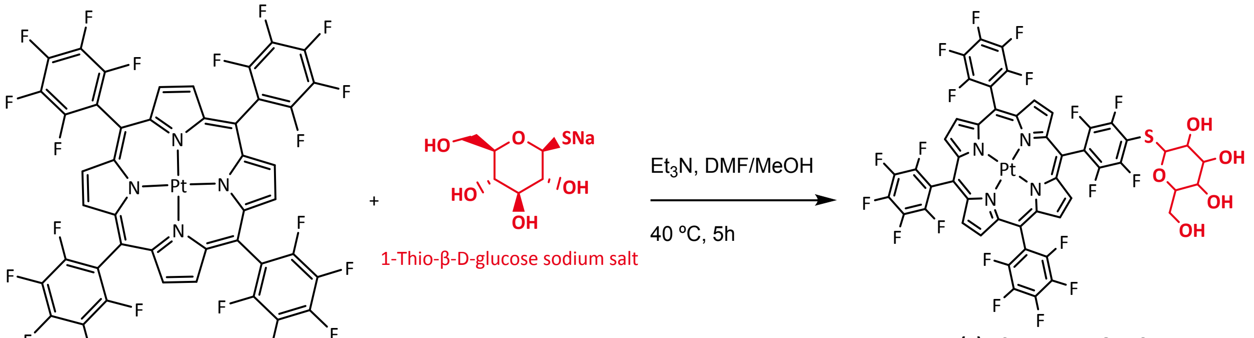

PtTFPP, Mw: 1167.63

(5) - $C_{50}H_{19}F_{19}N_4O_5PtS$
Mw: 1343.84

Pt2Glc$_1$ ***(6)*** was synthesized likewise using 2-Thioethyl-b-D-glucopyranoside (0.240 mg, 1 µmoles 0.31 mg yield 44.42%.

1.5. *PtcPEG$_3$1Glc* ***(7)****.* The mono-glycosylated compound **5** (0.67 mg, 500 nmoles) dissolved in 100µl of DMF was incubated with cPEG7-SH (2.3 mg, 5 µmoles), in the presence of TEA (1.63 µl, 10 µmoles) for 5h at 40°C, then purified by RP-HPLC (1.296 mg, yield 97.5%). Purity (HPLC): 92.7%. QToF high resolution mass spectrometry: m/z for $C_{107}H_{128}F_{16}N_4O_{35}PtS_4^{2-}$($[M-2H]^{2-}$) cacld 1402.07994, found 1402.0421. ^{1}H NMR (600 MHz, ($CDCl_3$, $CDCl_3$= 7.2 ppm) δ ppm= 2.47-2.51 (6H, m, C*H$_2$*-COOH), 3.3-3.96 (108H, m, O-(C*H$_2$*)$_{51}$ and 2',3',4',5',6'a,6'b-Glc*H*), 5.05 (1H, d, J=8.22 Hz, 1'-Glc*H*), 8.43 (8H, d, J=16.61 Hz, β-pyrrole*H*). ^{13}C NMR (600 MHz, $CDCl_3$, $CHCl_3$ =75.99) δ ppm= 33.28 (S-*C*H$_2$), 33.81 (COOH-*C*H$_2$), 65.43 (6'-Glc*C*), 69.04-69.86 (PEG-*C* chain and 4'-Glc*C*), 72.94 (2'-Glc*C*), 77.31 (3'-Glc*C*), 79.22 (5'-Glc*C*), 84.47 (1'-Glc*C*), 106.13 (meso*C*), 115.73 (1-Ph*C*), 118.3 (4-Ph*C*), 130.01 (β-pyrrole*C*), 139.97 (α-pyrrole*C*), 144.19-146.64 (3,5-Ph*C* and 2,6-Ph*C*), 173.30 (-*C*=OOH). ^{19}F NMR (300 MHz, CD_3OD) δ ppm= -131.84,-131.97 (2F, m, *meta*- 3,5-Ph*F*Glc), -133.41,-133.58 (6F, m, *meta*- 3,5-Ph*F*PEG), 136.72,-136.85 (2F, m, *ortho*- 2,6-Ph*F*Glc), 137.04,-137,24 (6F, m, *ortho*- 2,6-Ph*F*PEG). UV–vis (DMF, path length = 1.0 cm, 25°C): λ(nm)=393, 538, 506. FL (c = 0.2 µM, DMF, path length = 1.0 cm, λex = 390 nm, 25 °C): λ(nm) =649, 703.

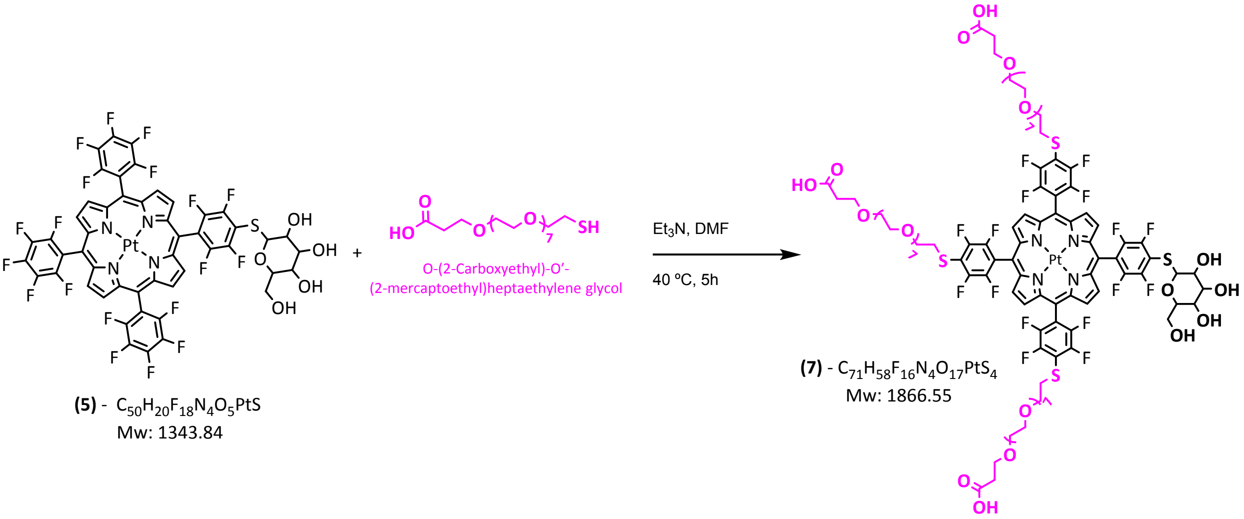

PtmPEG$_3$1Glc ***(8)****, PtcPEG$_3$2Glc* ***(9)****, PtmPEG$_3$2Glc* ***(10)*** were synthesized likewise using the mono-glycosylated compounds **5** (0.67 mg, 500 nmoles) or **6** (0.69 mg, 500 nmoles) dissolved in 100µl of DMF were incubated with cPEG7-SH (2.3 mg, 5 µmoles) or mPEG7-SH (1.78 mg, 5 µmoles), obtaining 0.77 mg, yield 65.15% (**8**); 1.269 mg, yield 93.9% (**9**); 0.86 mg, yield 71.76% (**10**). Purity (HPLC): 84.2% ***(8)***, 93.9% ***(9)***, 97.2% ***(10)***.

1.6. *PtCA$_3$1Glc (**11**),* The mono-glycosylated compounds **5** (0.67 mg, 500 nmoles) dissolved in 100 µl of DMF were incubated for 3h at 40°C with 2-(Boc-amino)ethanethiol (1 µl, 5 µmoles) in the presence of TEA (1.63 µl, 10 µmoles)This was followed by TEA neutralisation with 1 molar equivalent of HCl and purification by RP-HPLC (yield 90-95%).. Deprotection was carried out by dissolving the dried product in acetonitrile: methanol (3:2) containing 1M HCl and overnight stirring at 55°C. The acid was then neutralized by 1 molar equivalent of TEA, the reaction was concentrated and the residue dissolved in MeOH for purification (0.655 mg, yield 84.12%). Purity (HPLC): 98.8%. QToF high resolution mass spectrometry: m/z for $C_{56}H_{40}F_{16}N_7O_5PtS_4^{3+}$ ($[M+3H]^{3+}$) cacld 506.044279, found 506.0499. ^{1}H NMR (600 MHz, (CD_3OD, CH_3OH= 3.33ppm) δ ppm= 3.43-3.56 (16H, m, S-C*H$_2$*-C*H$_2$* and 4’,2’,3’,5’-Glc*H*), 3.76 (1H, dd, J1,2= 12.1, 6.56, 6’-Glc*H*), 4.02 (1H, dd, J1,2=11.88, 1.84, 6’-Glc*H*) 5.17- 5.22 (1H, m, 1’-Glc*H*), 9.11-9.17 (8H, m, β-pyrrole*H*). ^{13}C NMR (600 MHz, CD_3OD, CH_3OH =49.0) δ ppm= 33.01 (N-*C*H$_2$), 40.79 (S-*C*H$_2$), 63.11 (6’-Glc*C*), 71.74 (4’-Glc*C*), 75.96 (2’-Glc*C*), 79.74 (3’-Glc*C*), 82.77 (5’-Glc*C*), 86.68 (6’-Glc*C*), 108.28 (meso*C*), 115.76 (1-Ph*C*), 121.83 (4-Ph*C*), 132.74 (β-pyrrole*C*), 142.7 (α-pyrrole*C*), 146.85-149.62 (3,5-Ph*C* and 2,6-Ph*C*). ^{19}F NMR (300 MHz, CD_3OD) δ ppm= -134.87 (2F, dd, $J^{1,2}$= 24.45, 11.70, *meta*- 3,5-Ph*F*Glc), -135.27, -135.40 (6F, m, *meta*- 3,5-Ph*F*CA), 139.29, -139.42 (6F, m, *ortho*- 2,6-Ph*F*Glc), 140.62 (2F, dd, $J^{1,2}$= 24.32, 11.73, *ortho*- 2,6-Ph*F*CA). UV–vis (DMF, path length = 1.0 cm, 25°C): λ(nm)=393, 538, 506. FL (c = 0.2µM, DMF, path length = 1.0 cm, λex = 390 nm, 25 °C): λ(nm) =649,703.

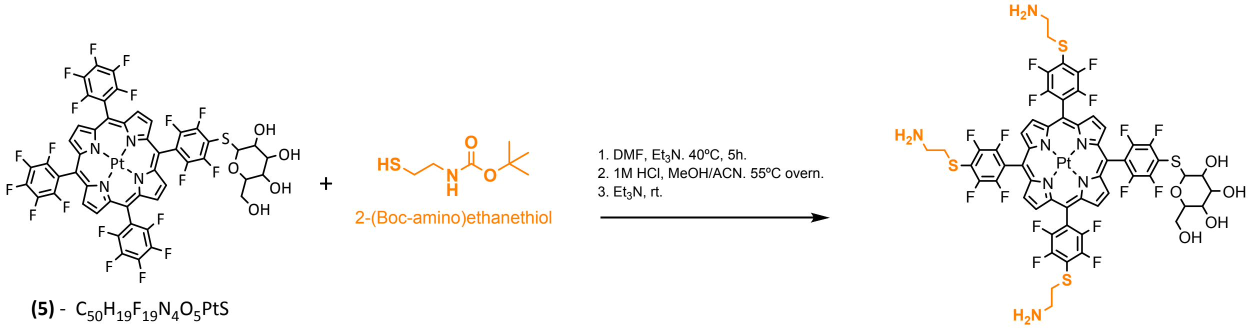

(5) - $C_{50}H_{19}F_{19}N_4O_5PtS$
Mw: 1343.84

(11) - $C_{56}H_{37}F_{16}N_7O_5PtS_4$
Mw: 1515.25

*PtCA$_3$2Glc (**12**).* was synthesized likewise using the mono-glycosylated compound **6** (0.69 mg, 500 nmoles) obtaining 0.282 mg, yield 35.18% (**12**). Purity (HPLC): 100%.

2. NMR and chemical structures

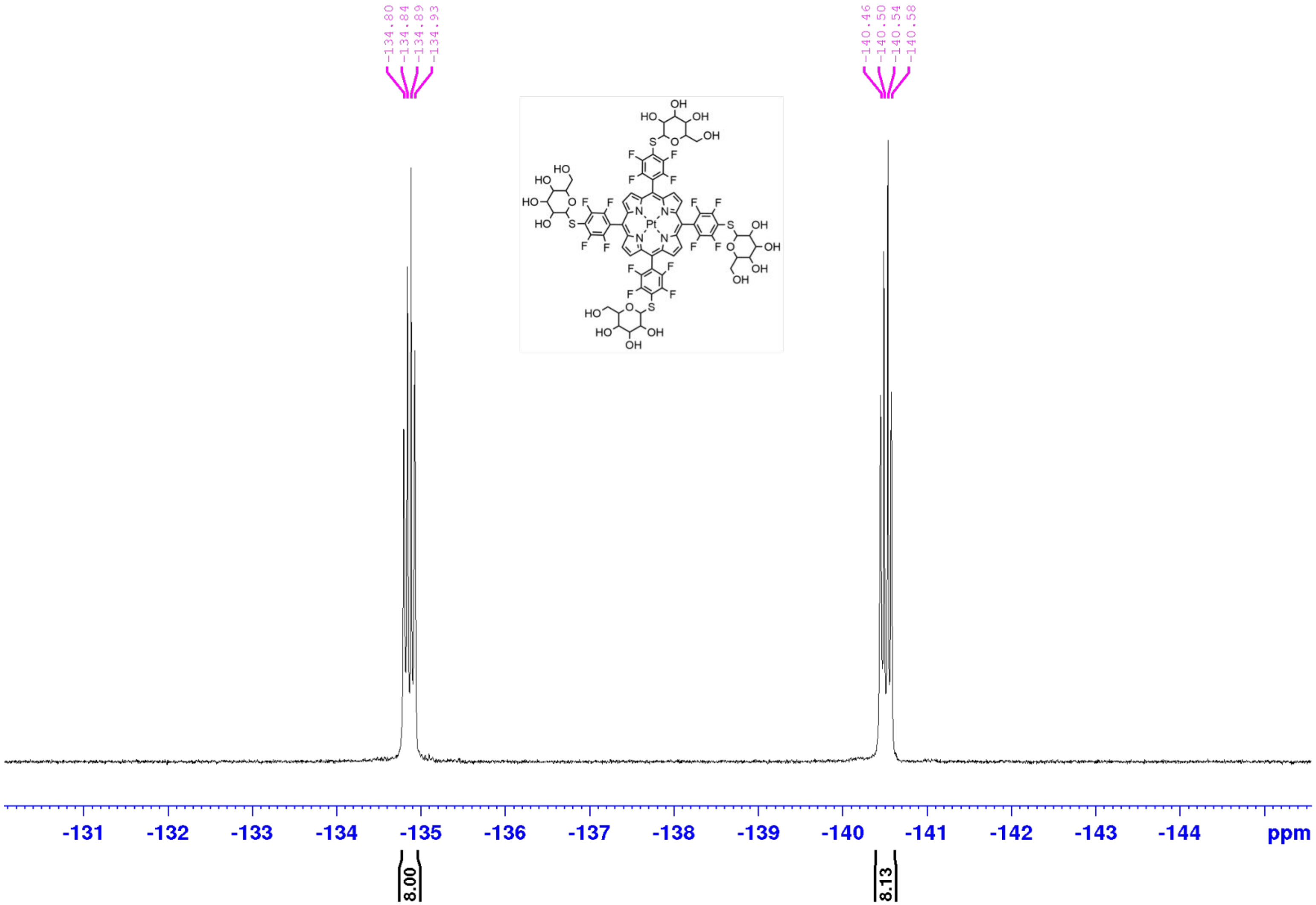

Spectrum 1: ^{19}F NMR **Pt1Glc$_4$** (1) in CD_3OD (300 MHz)

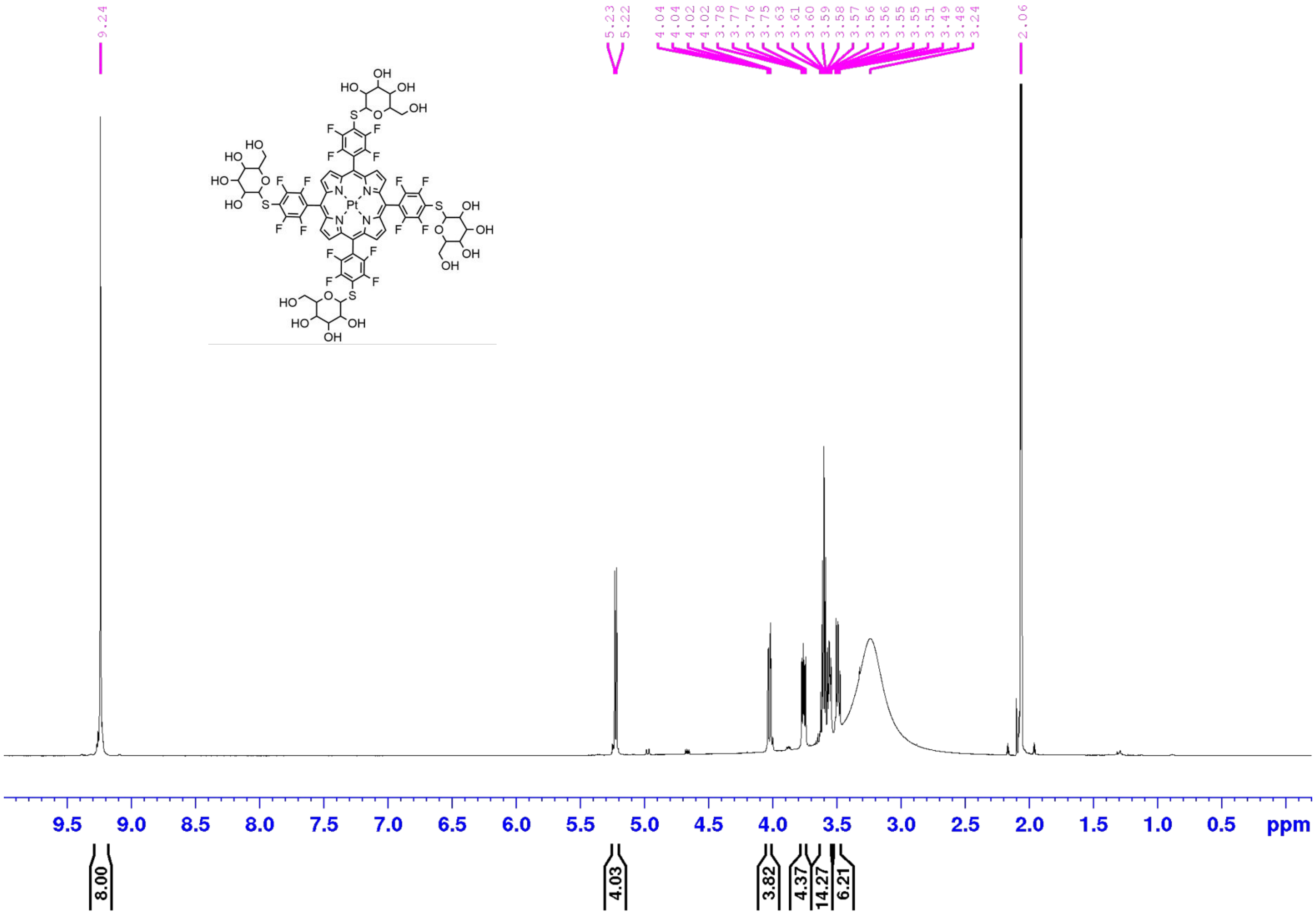

Spectrum 2: ^{1}H NMR **Pt1Glc$_4$** (1) in $(CD_3)_2CO$ (600 MHz)

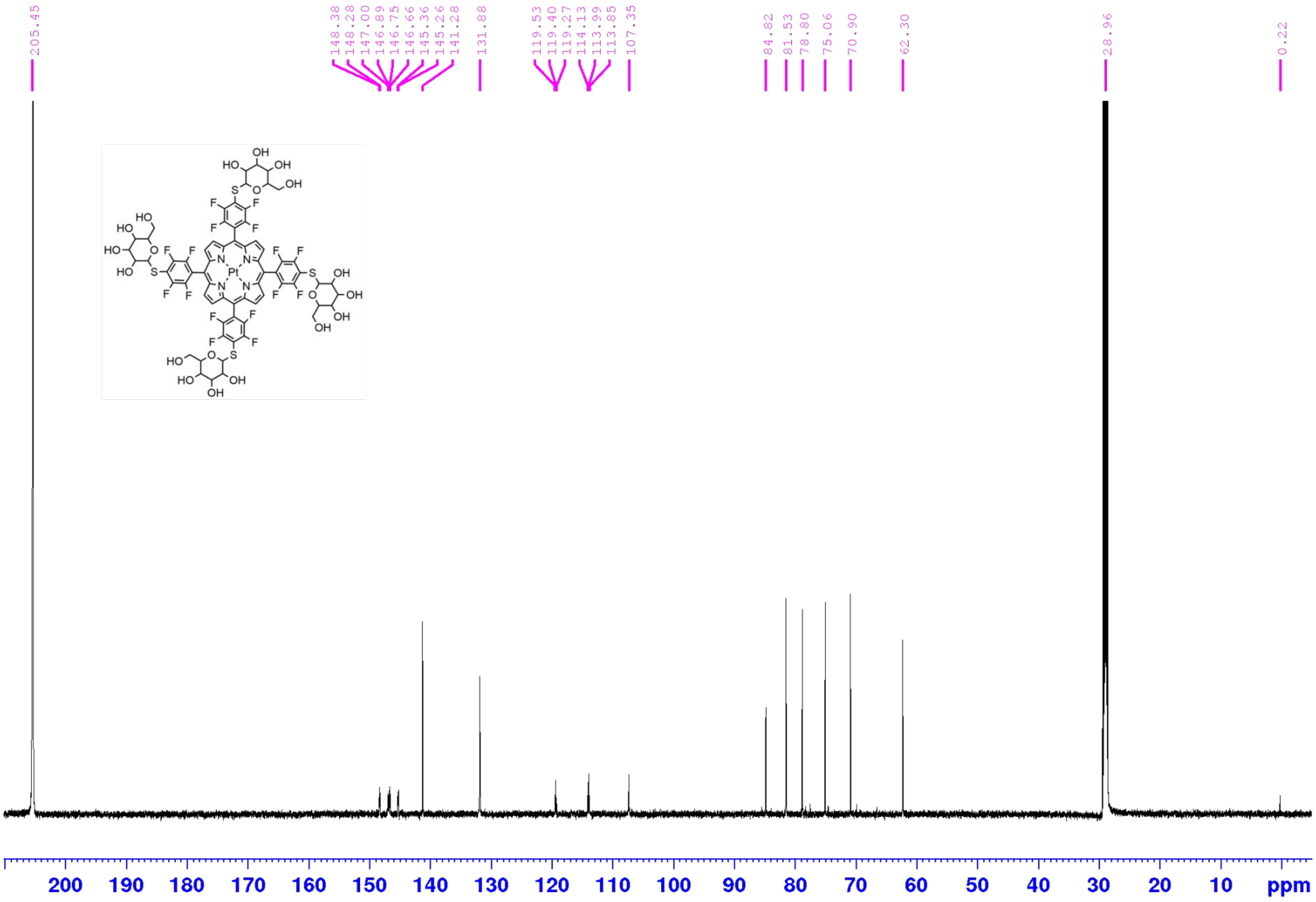

Spectrum 3: ^{13}C NMR **Pt1Glc$_4$** (1) in $(CD_3)_2CO$ (600 MHz)

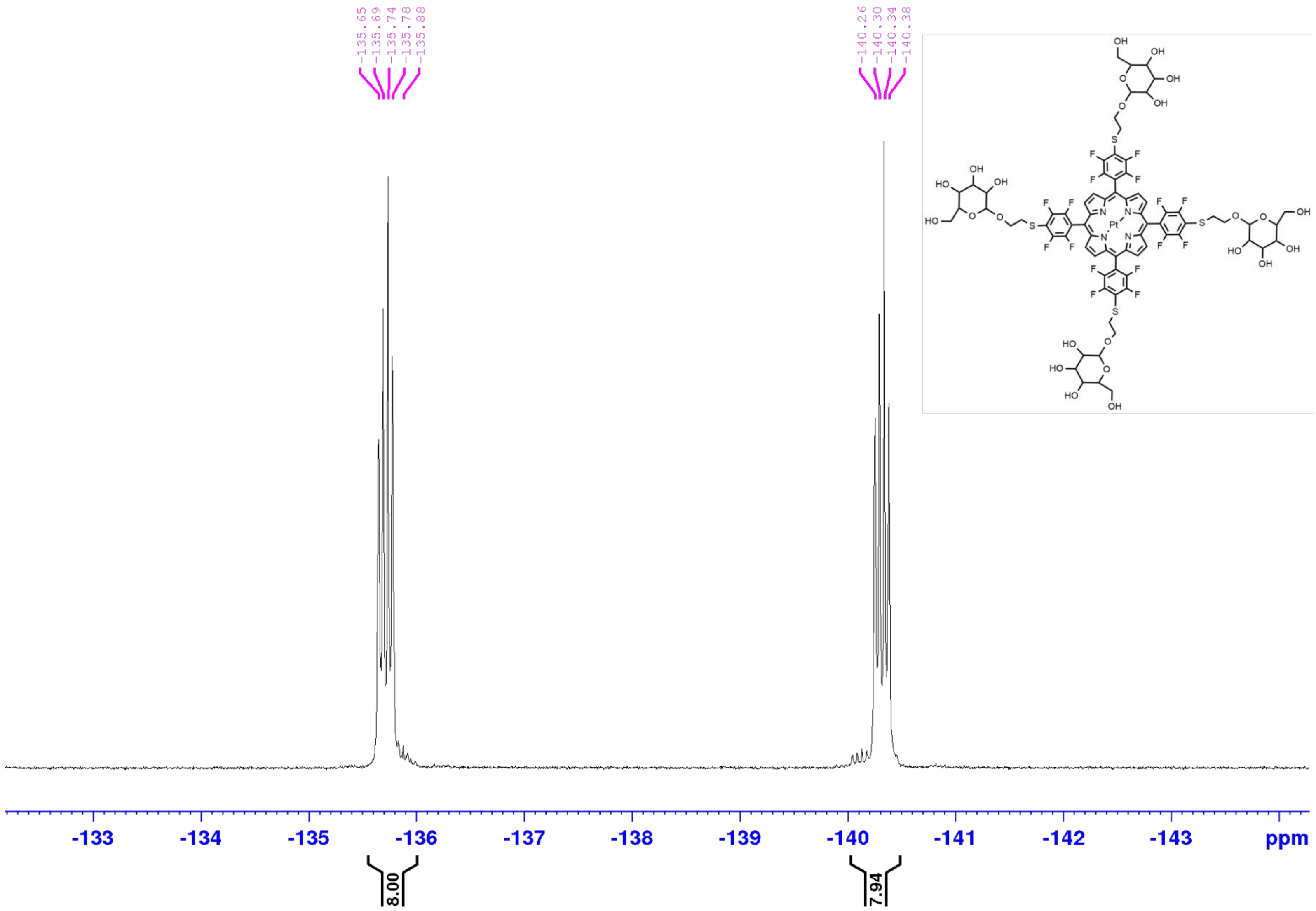

Spectrum 4: ^{19}F NMR **Pt2Glc$_4$** (2) in CD_3DO (300 MHz)

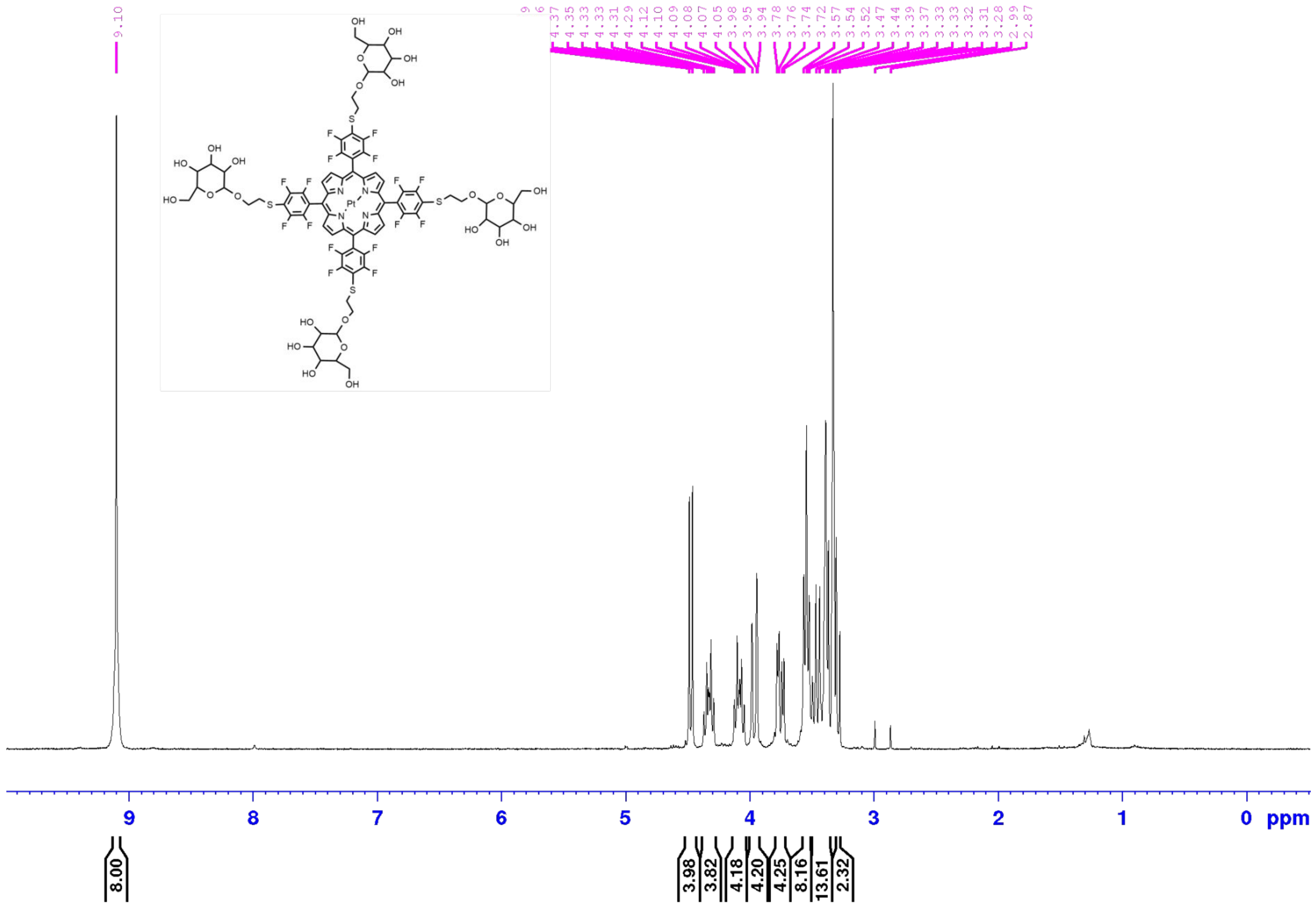

Spectrum 5: ^{1}H NMR **Pt2Glc$_4$** (2) in CD_3OD and water suppression (300 MHz)

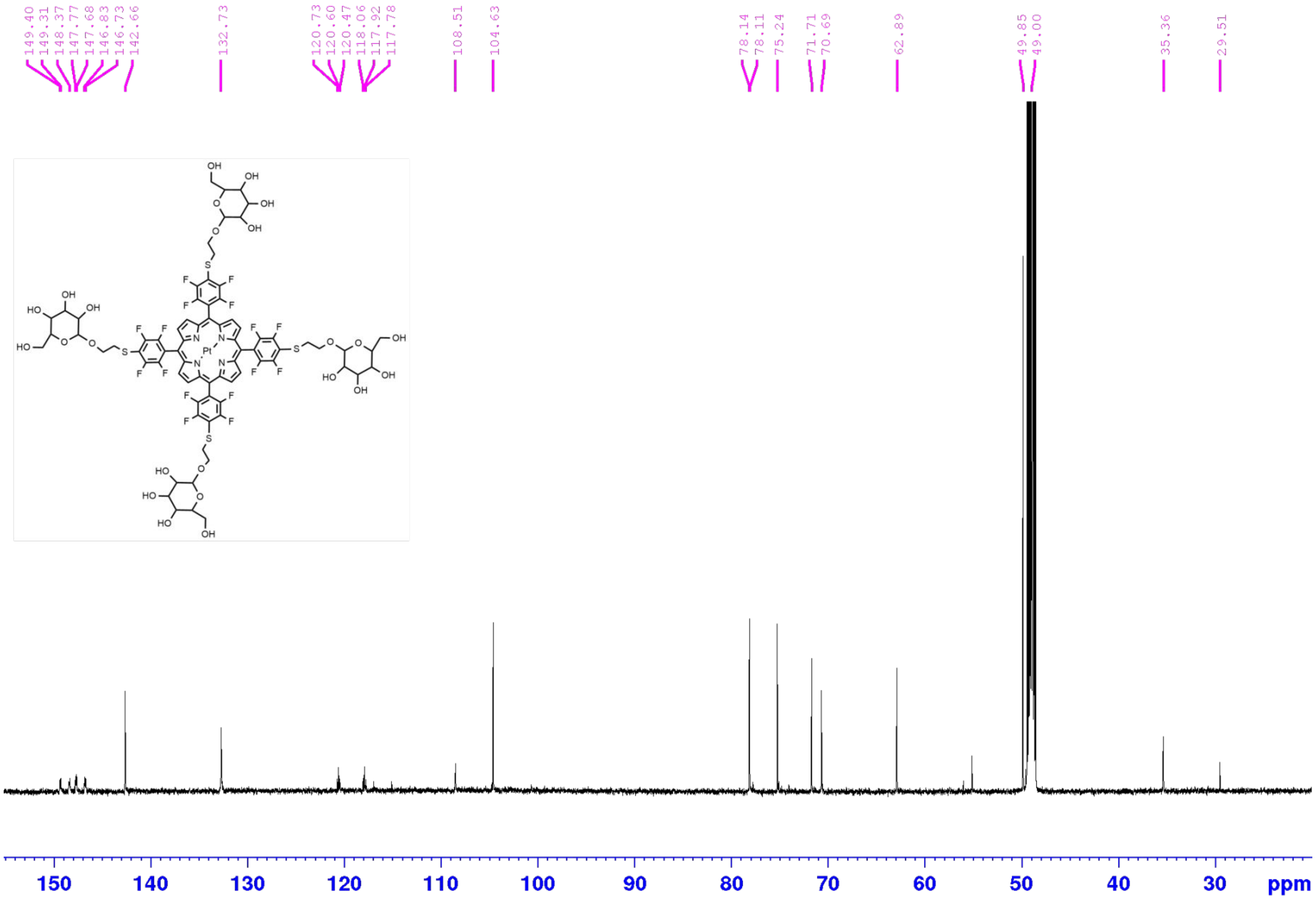

Spectrum 6: ^{13}C NMR **Pt2Glc$_4$** (2) in CD_3OD (600 MHz)

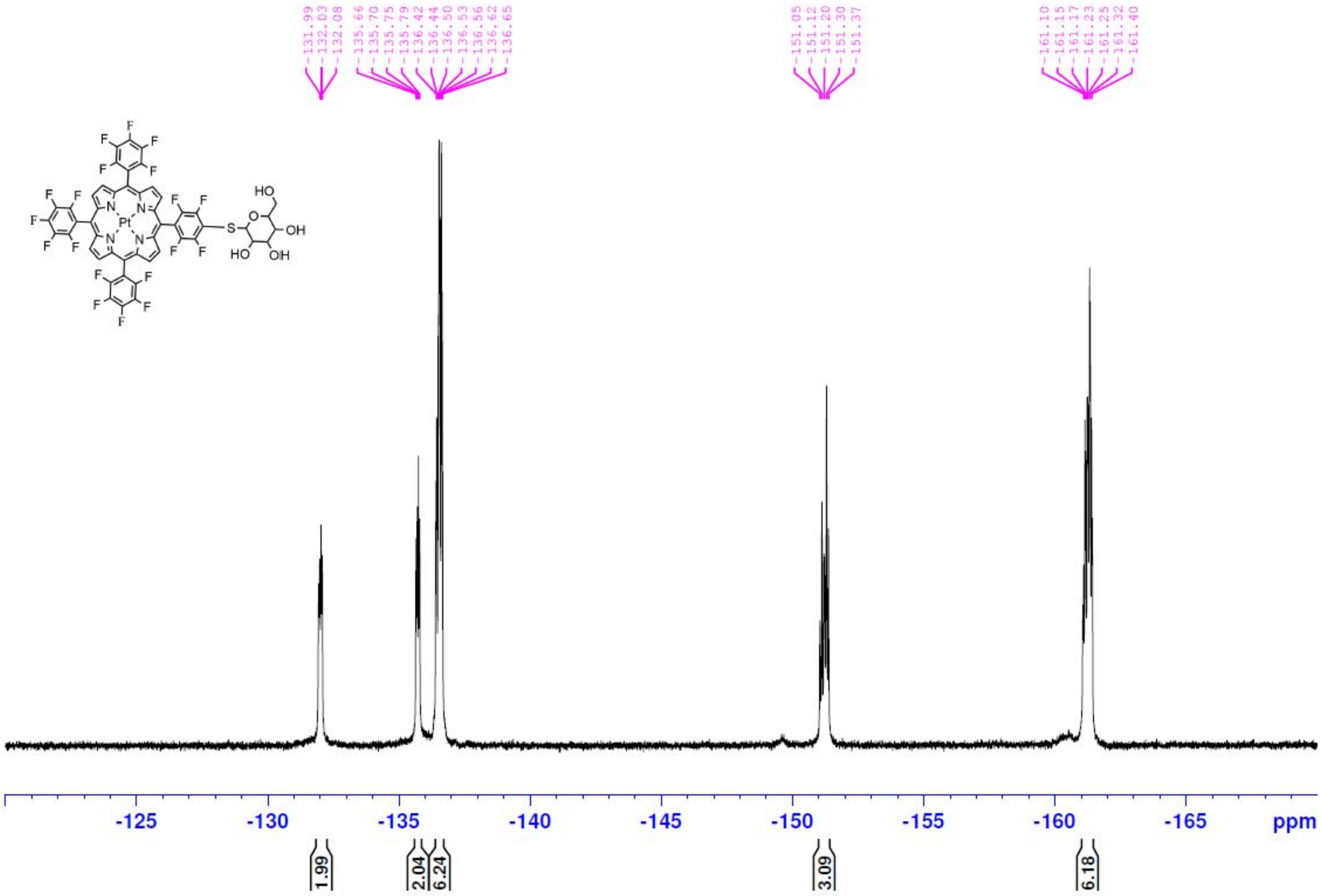

Spectrum 7: ^{19}F NMR **Pt1Glc$_1$** (5) in $CDCl_3$ (300 MHz)

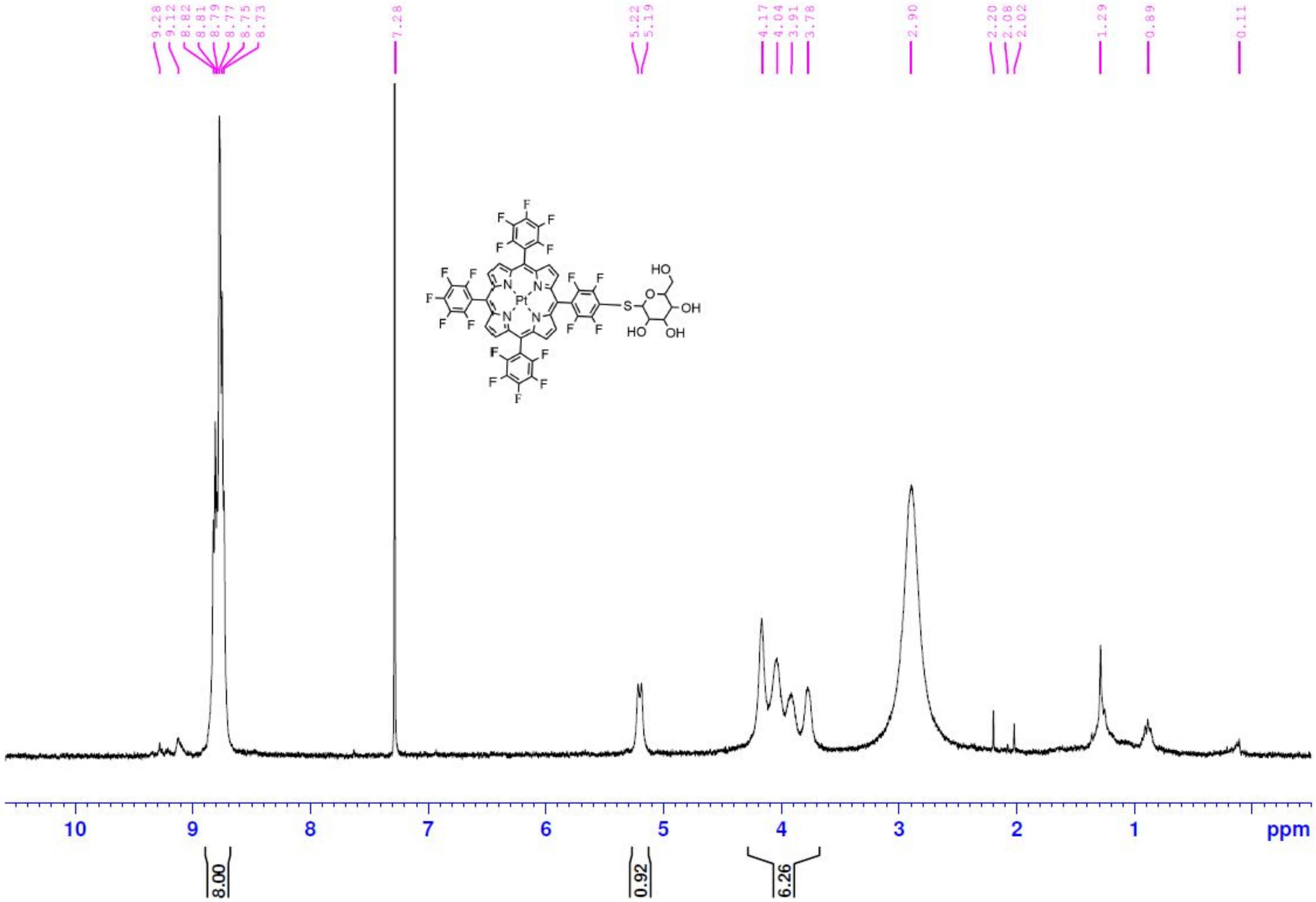

Spectrum 8: ^{1}H NMR **Pt1Glc$_1$** (5) in $CDCl_3$ (300 MHz)

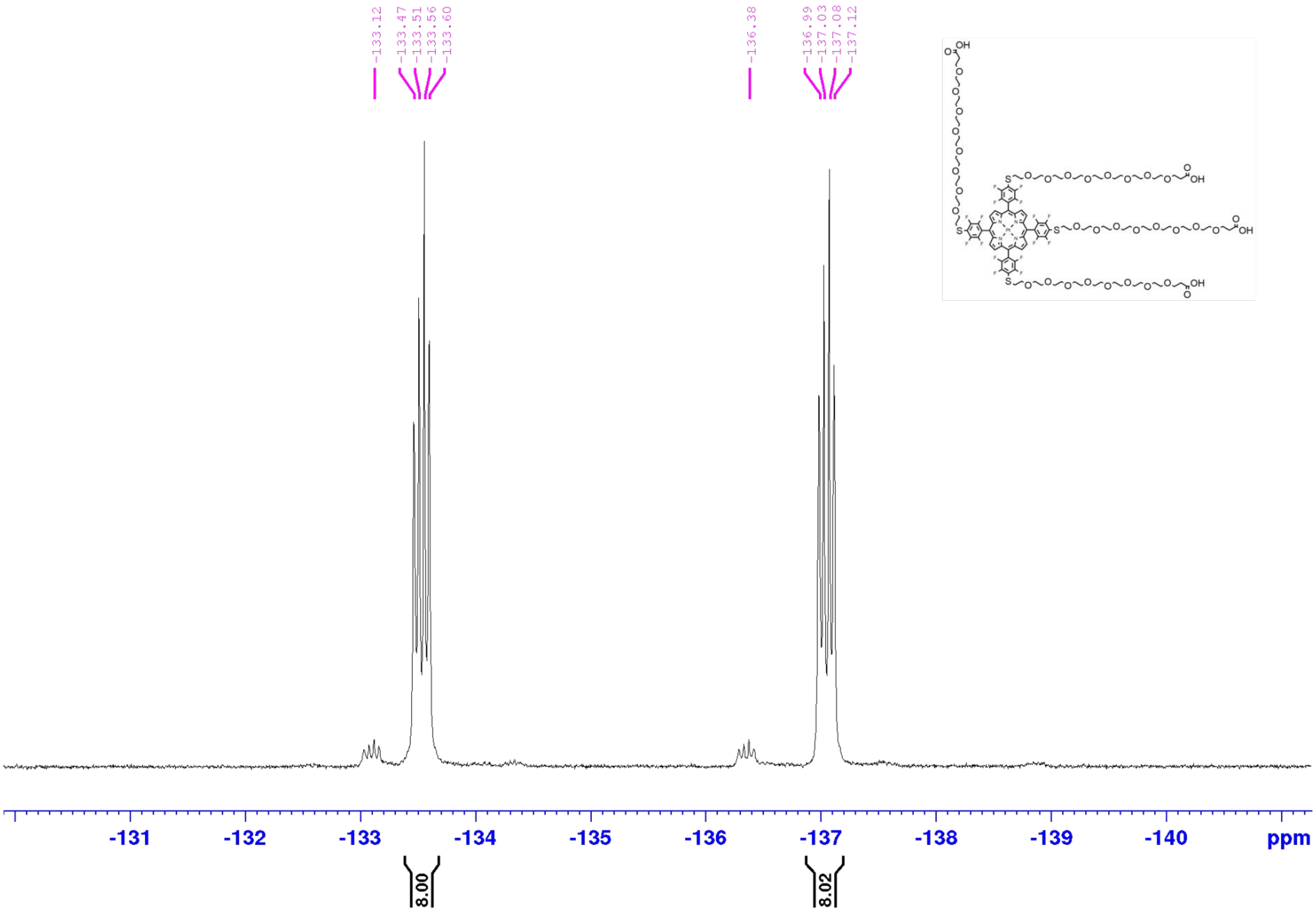

Spectrum 9: ^{19}F NMR **PtcPEG$_4$** (3) in $CDCl_3$ (300 MHz)

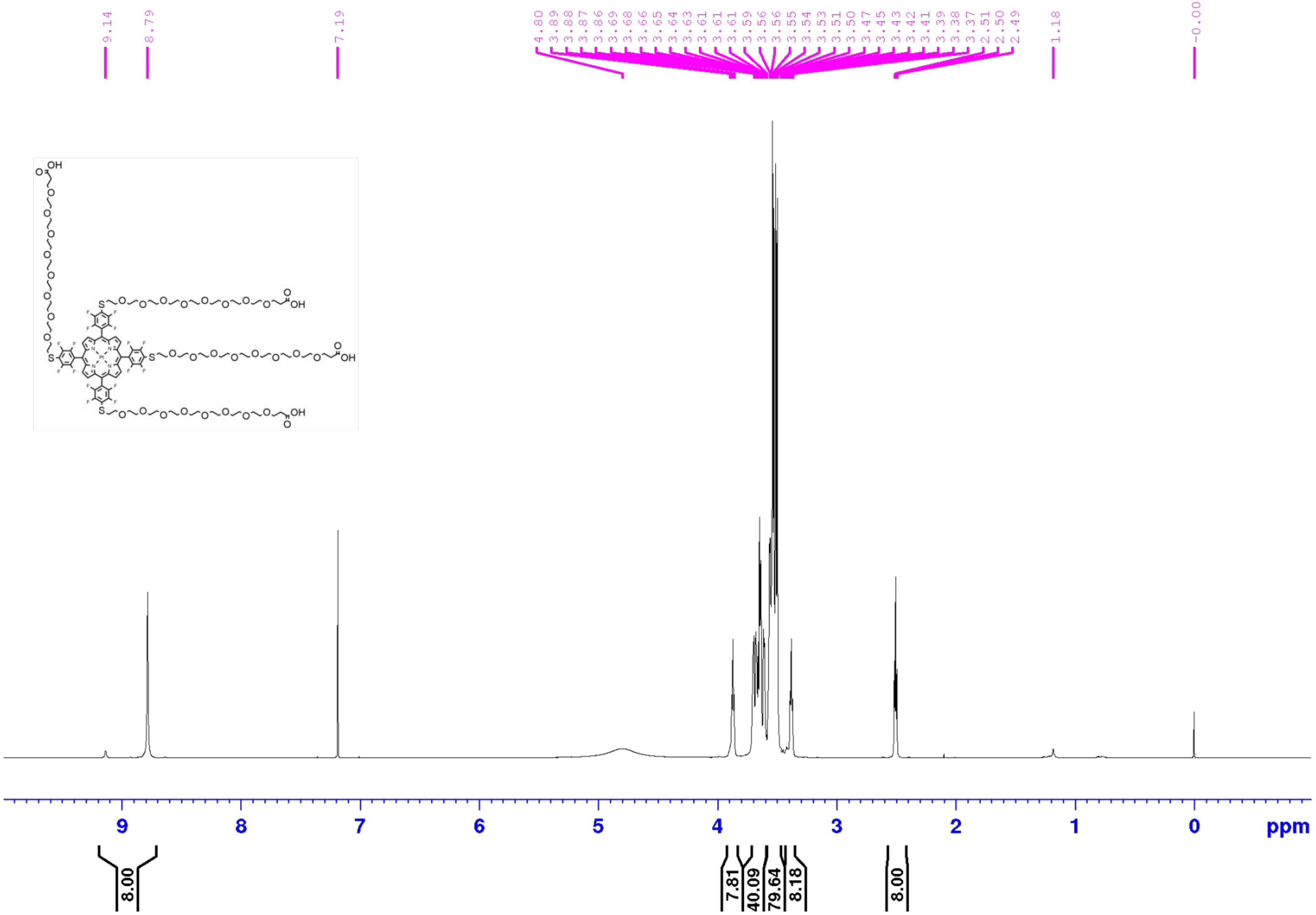

Spectrum 10: ^{1}H NMR **PtcPEG$_4$** (3) in $CDCl_3$ (600 MHz)

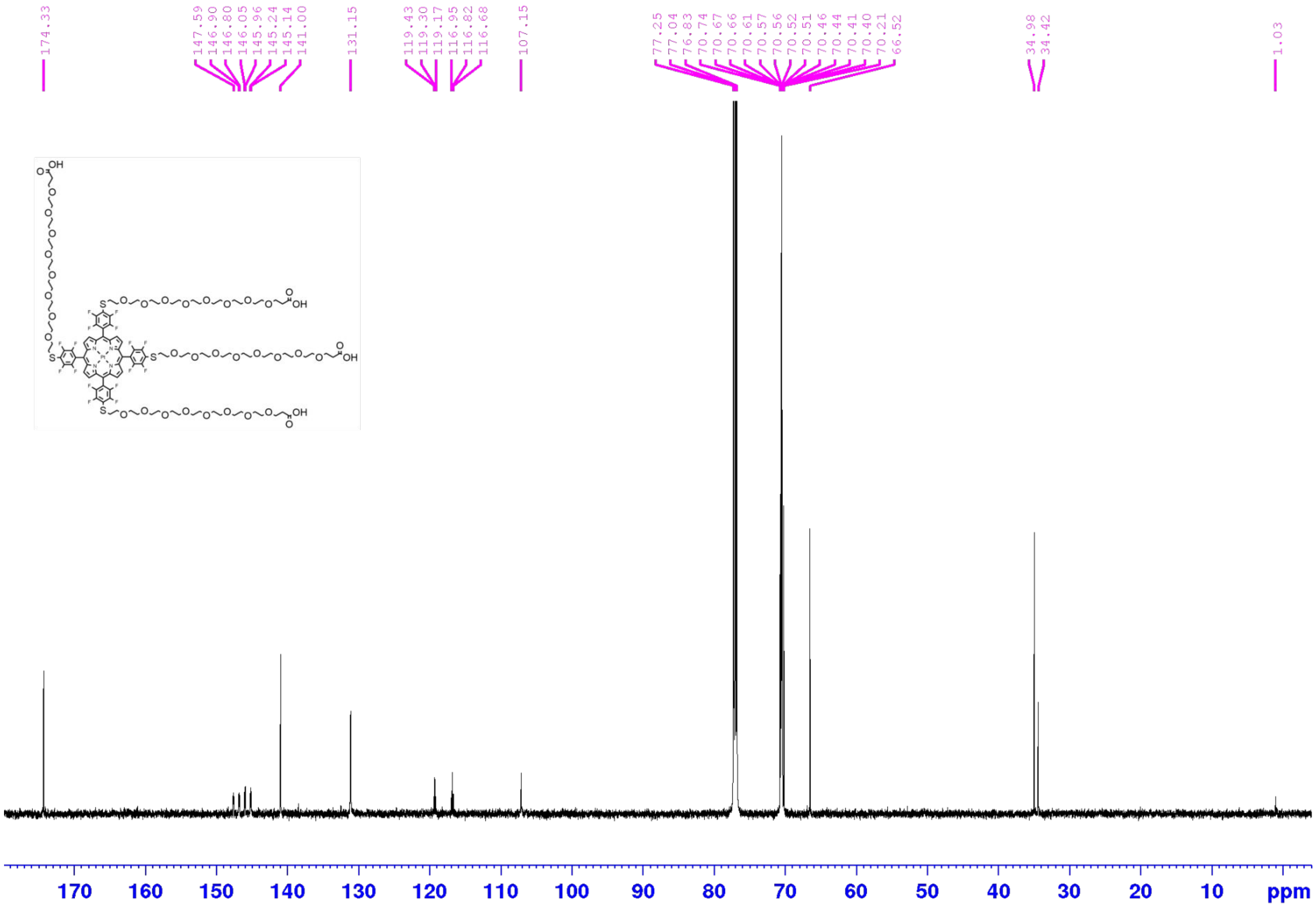

Spectrum 11: ^{13}C NMR **PtcPEG$_4$** (3) in $CDCl_3$ (600 MHz)

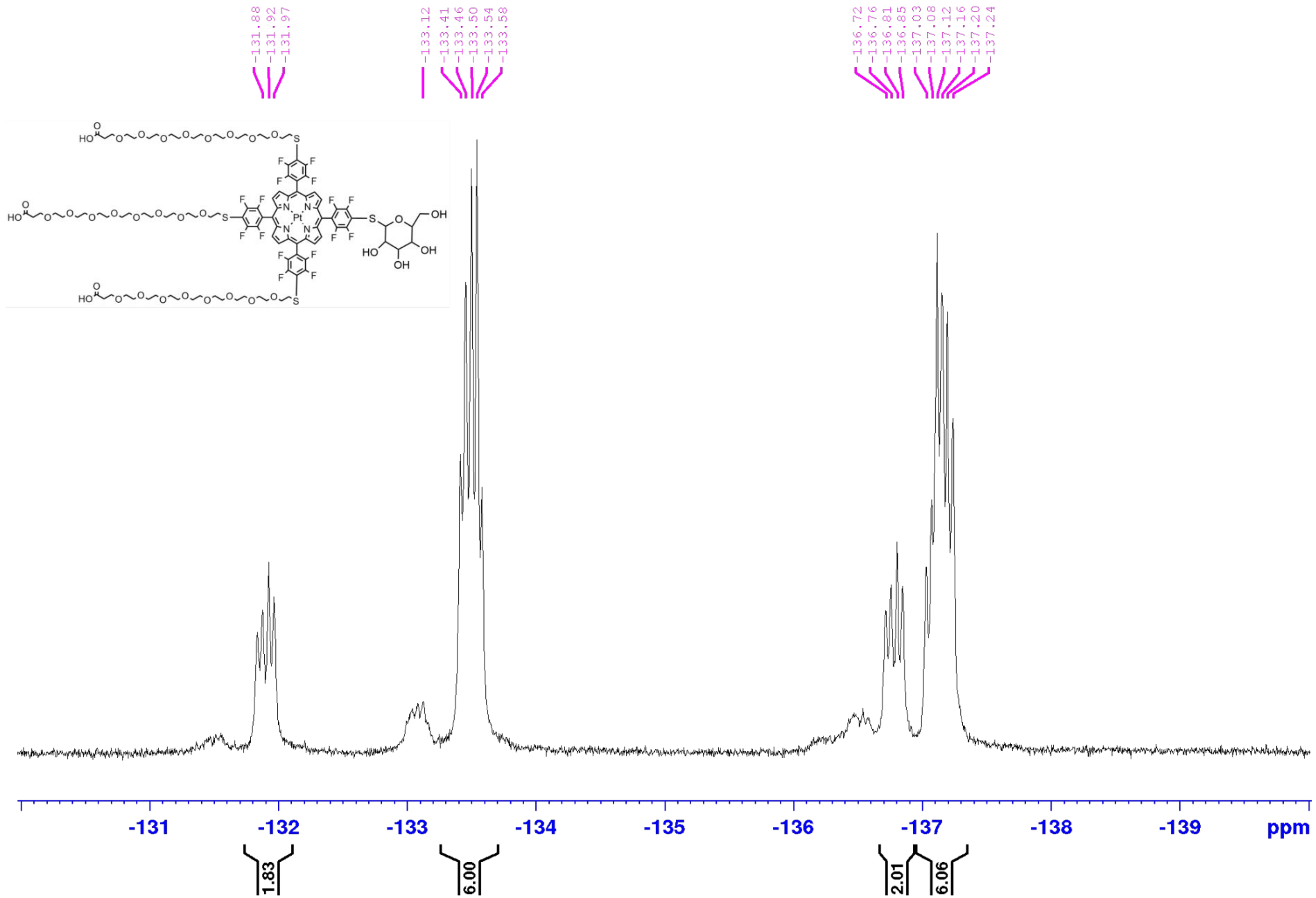

Spectrum 12: ^{19}F NMR **PtcPEG$_3$1Glc** (7) in $CDCl_3$ (300 MHz)

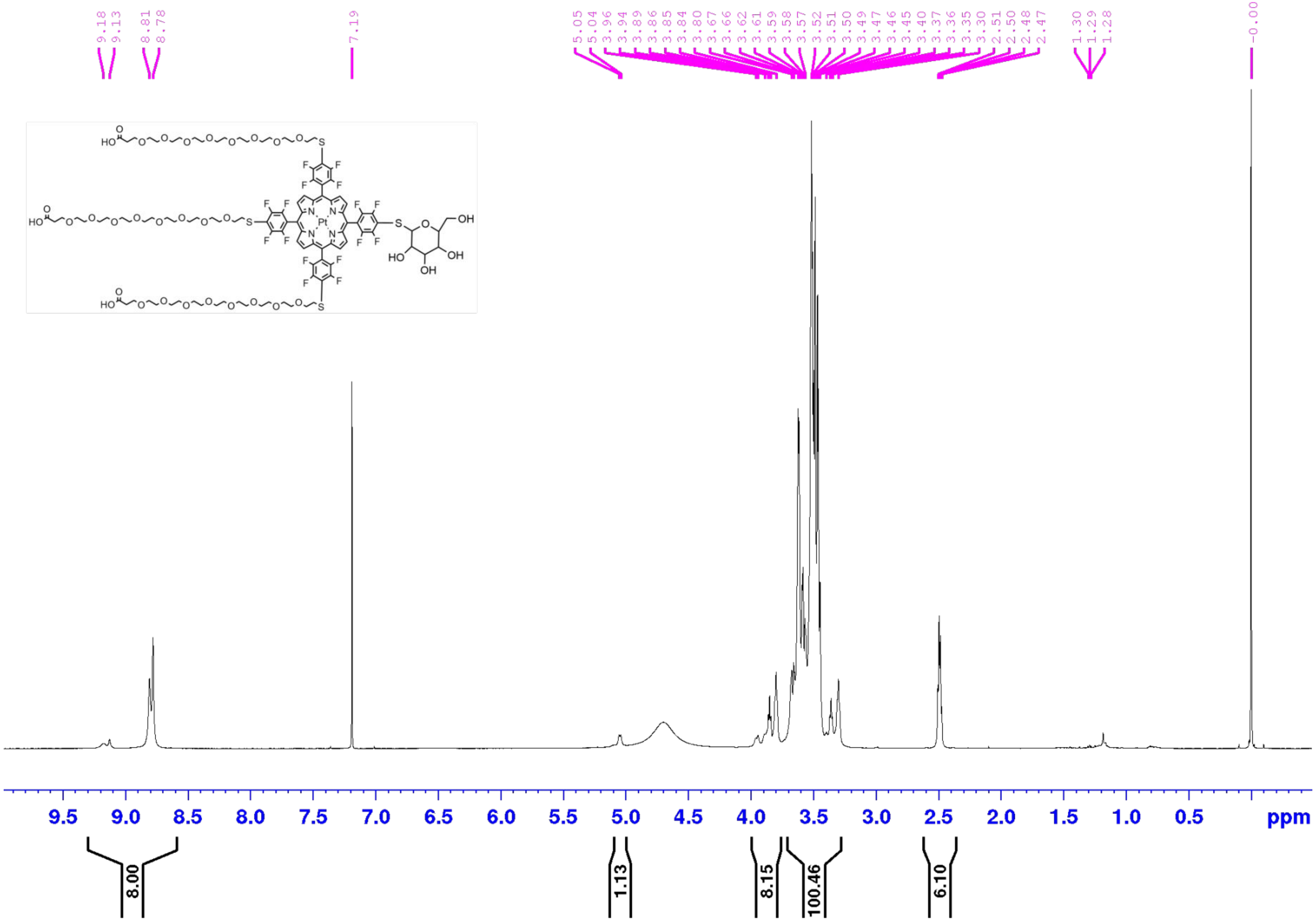

Spectrum 13: ^{1}H NMR **PtcPEG$_3$1Glc** (7) in $CDCl_3$ (600 MHz)

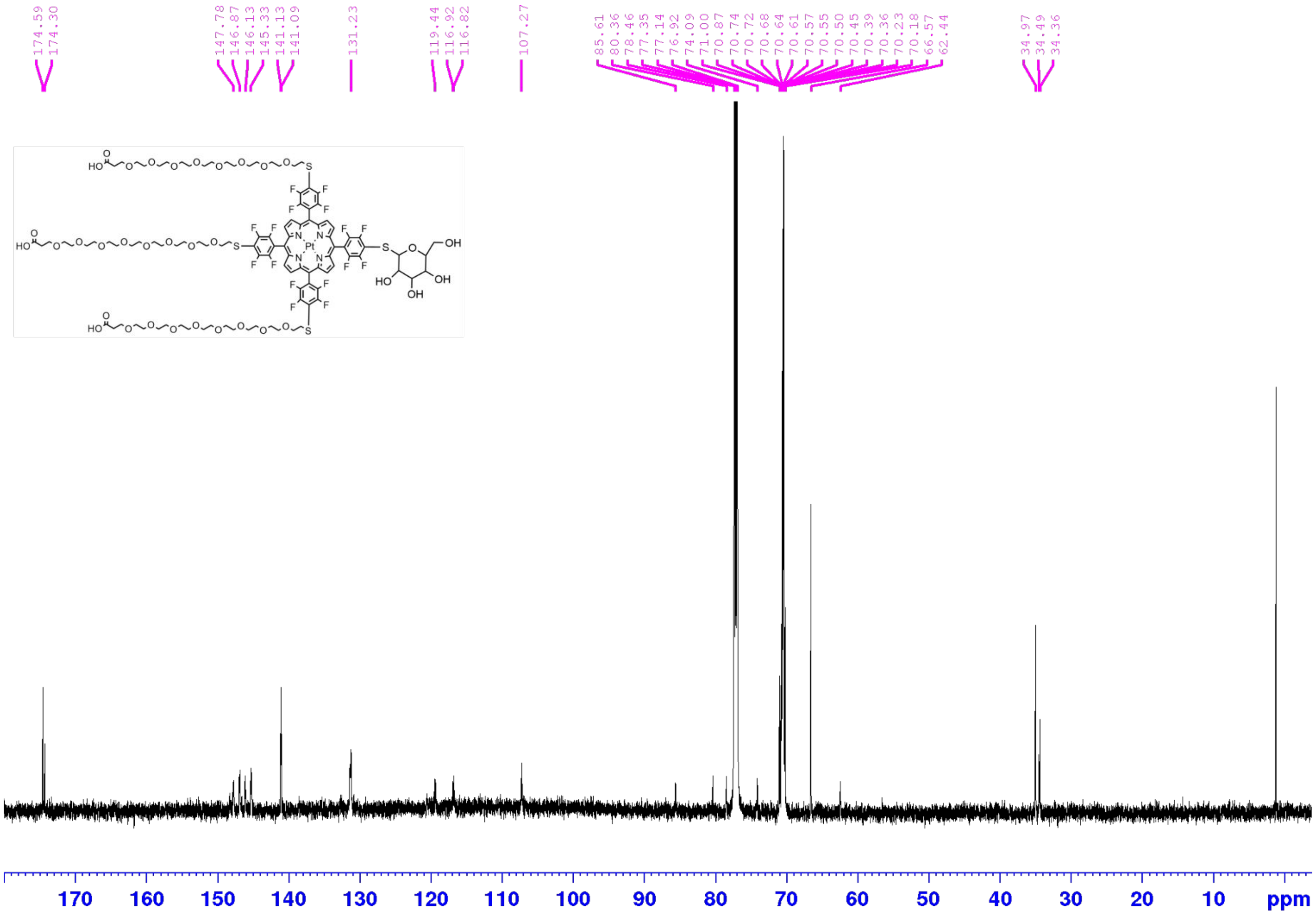

Spectrum 14: ^{13}C NMR **PtcPEG$_3$1Glc** (7) in $CDCl_3$ (600 MHz)

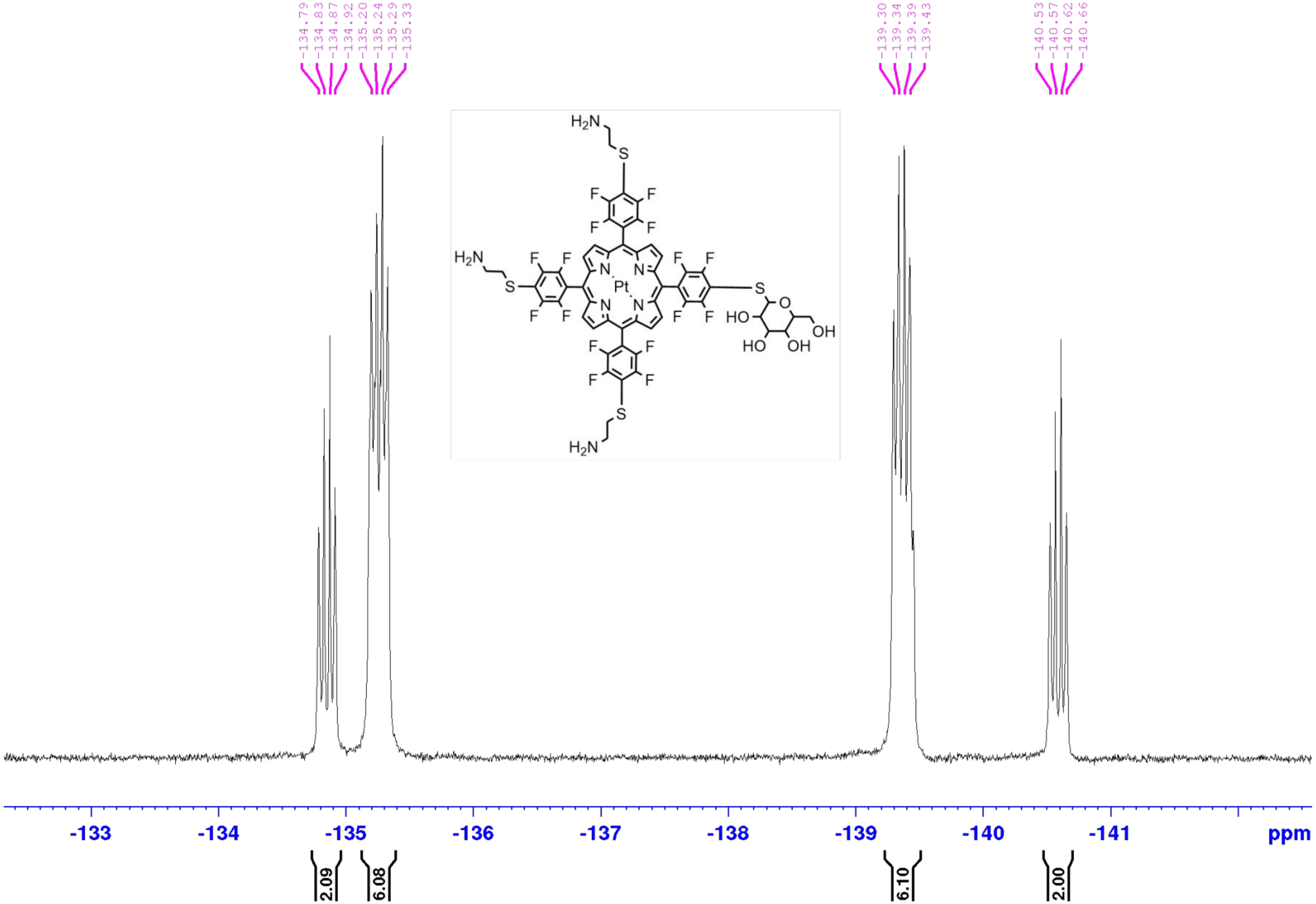

Spectrum 15 : ^{19}F NMR **PtCA$_3$1Glc** (11) in CD_3OD (600 MHz)

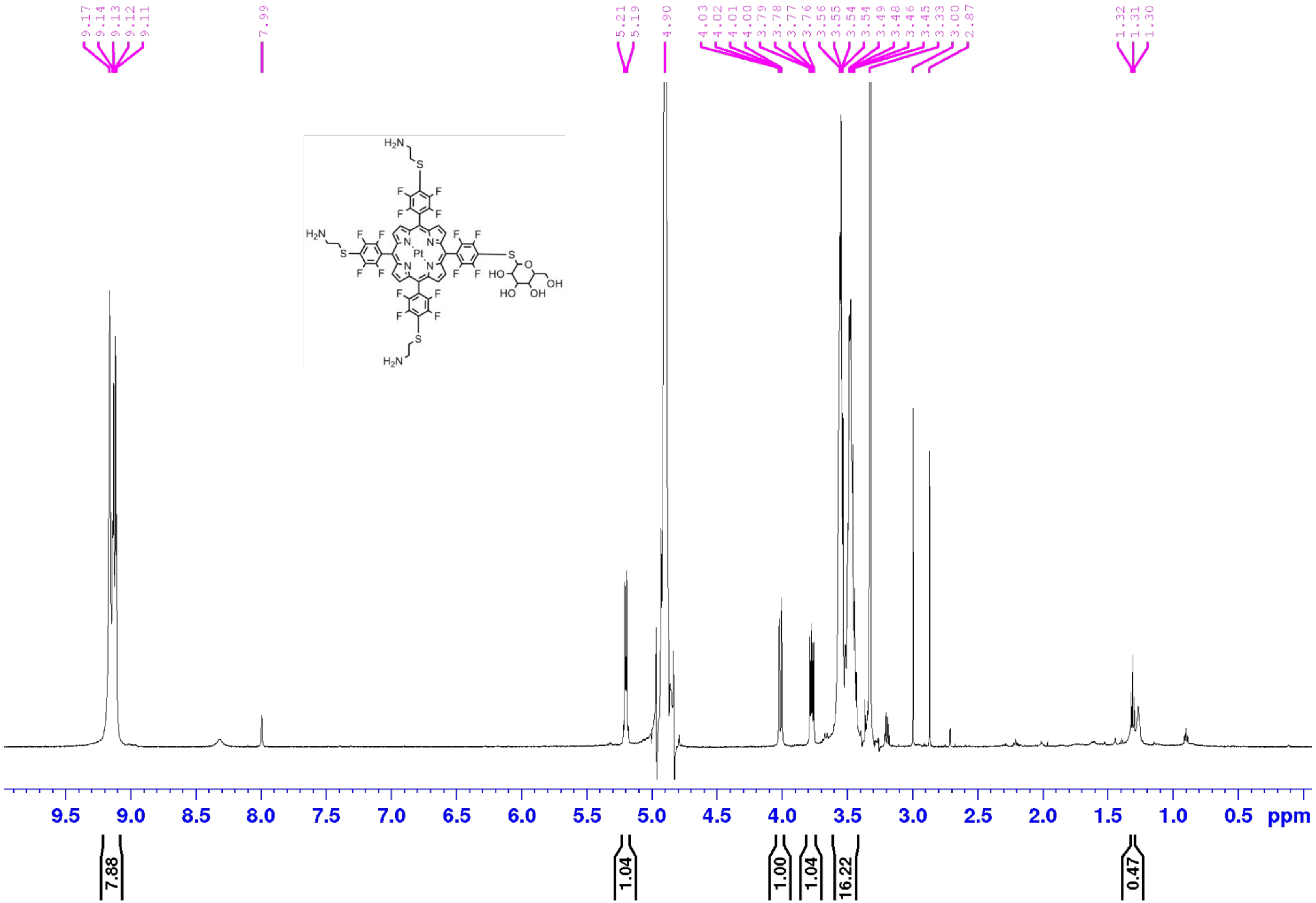

Spectrum 16: ^{1}H NMR **$PtCA_31Glc$** (11) in CD_3OD (600 MHz)

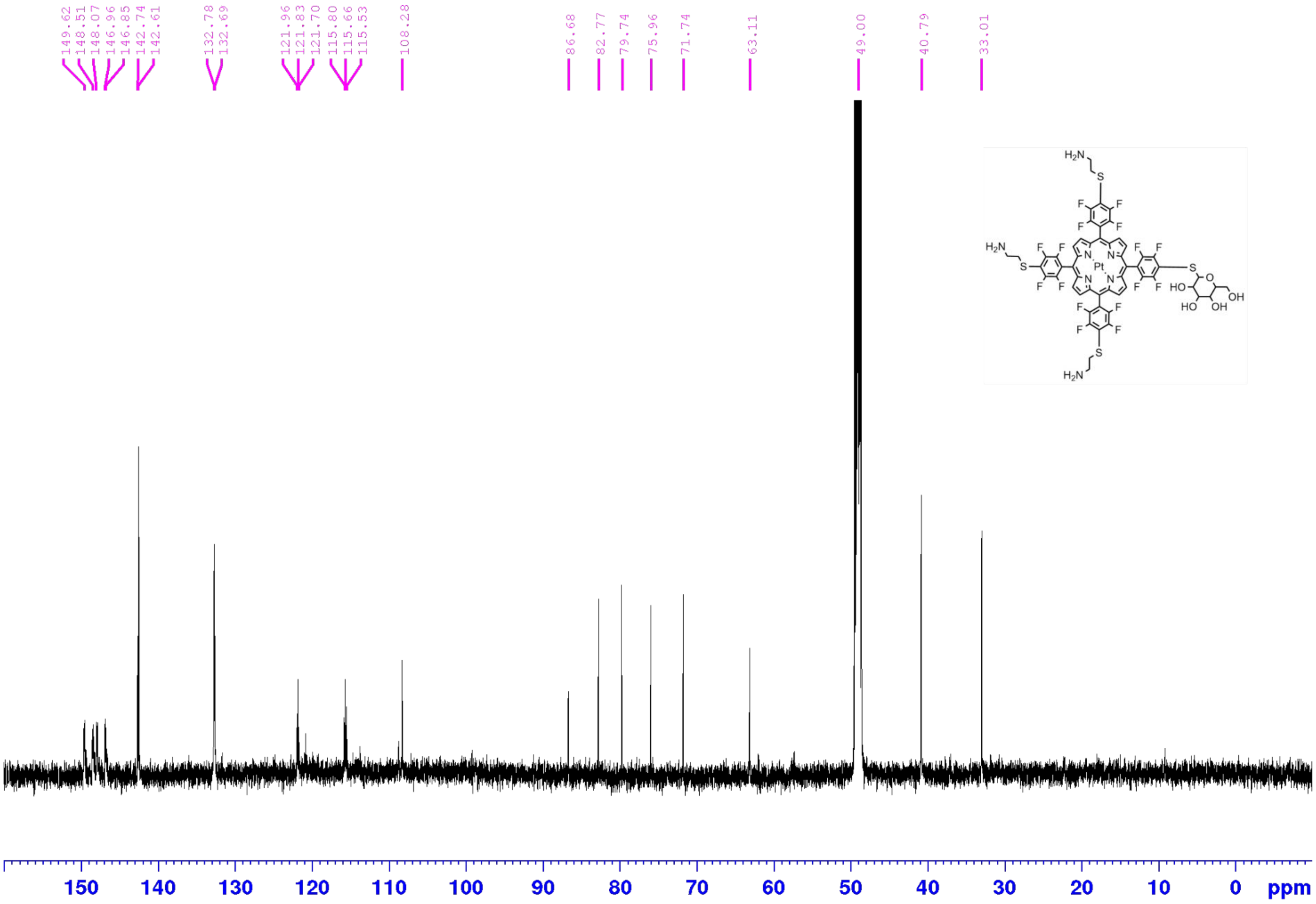

Spectrum 17: ^{13}C NMR **PtCA$_3$1Glc** (11) in CD_3OD (600 MHz)

3. Mass Spectra

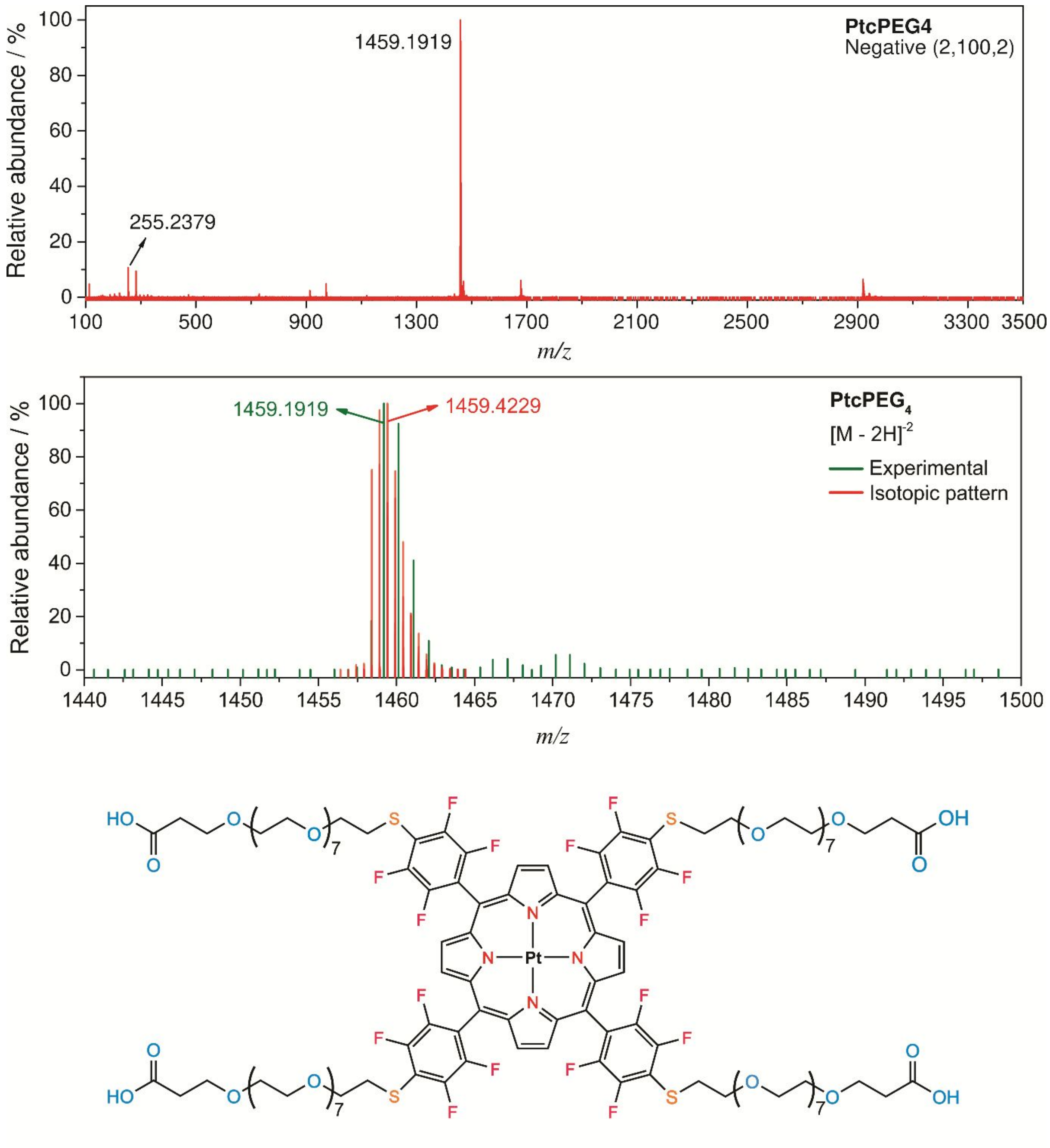

$C_{120}H_{154}F_{16}N_4O_{40}PtS_4$
100% r.a. *m/z*: 1459.42

Spectra 18: HR-ESI Q-TOF mass spectra and isotopic pattern of the PtcPEG$_4$ compound.

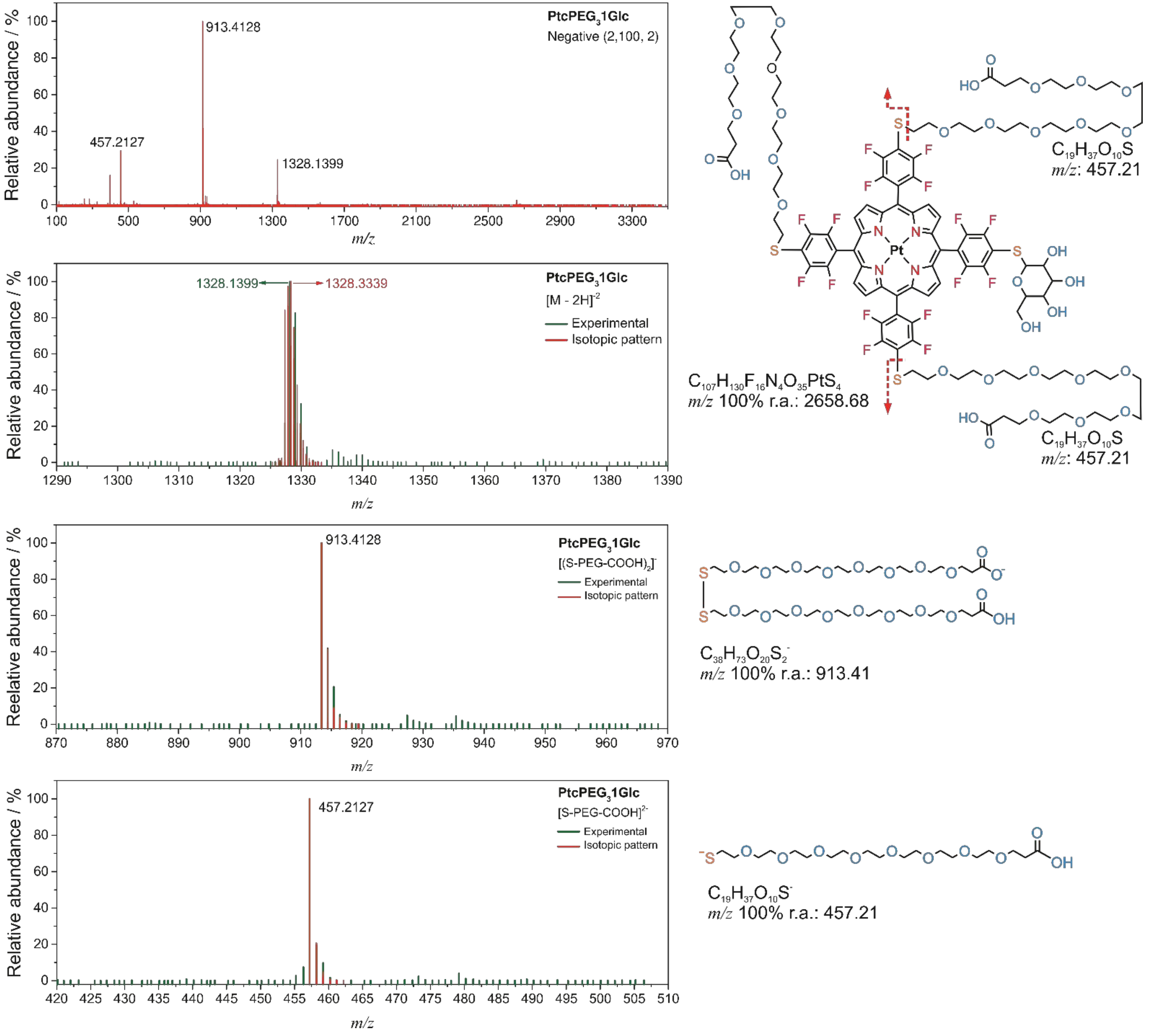

Spectra 19: HR-ESI Q-TOF mass spectra and isotopic pattern of the PtcPEG$_3$1Glc compound.

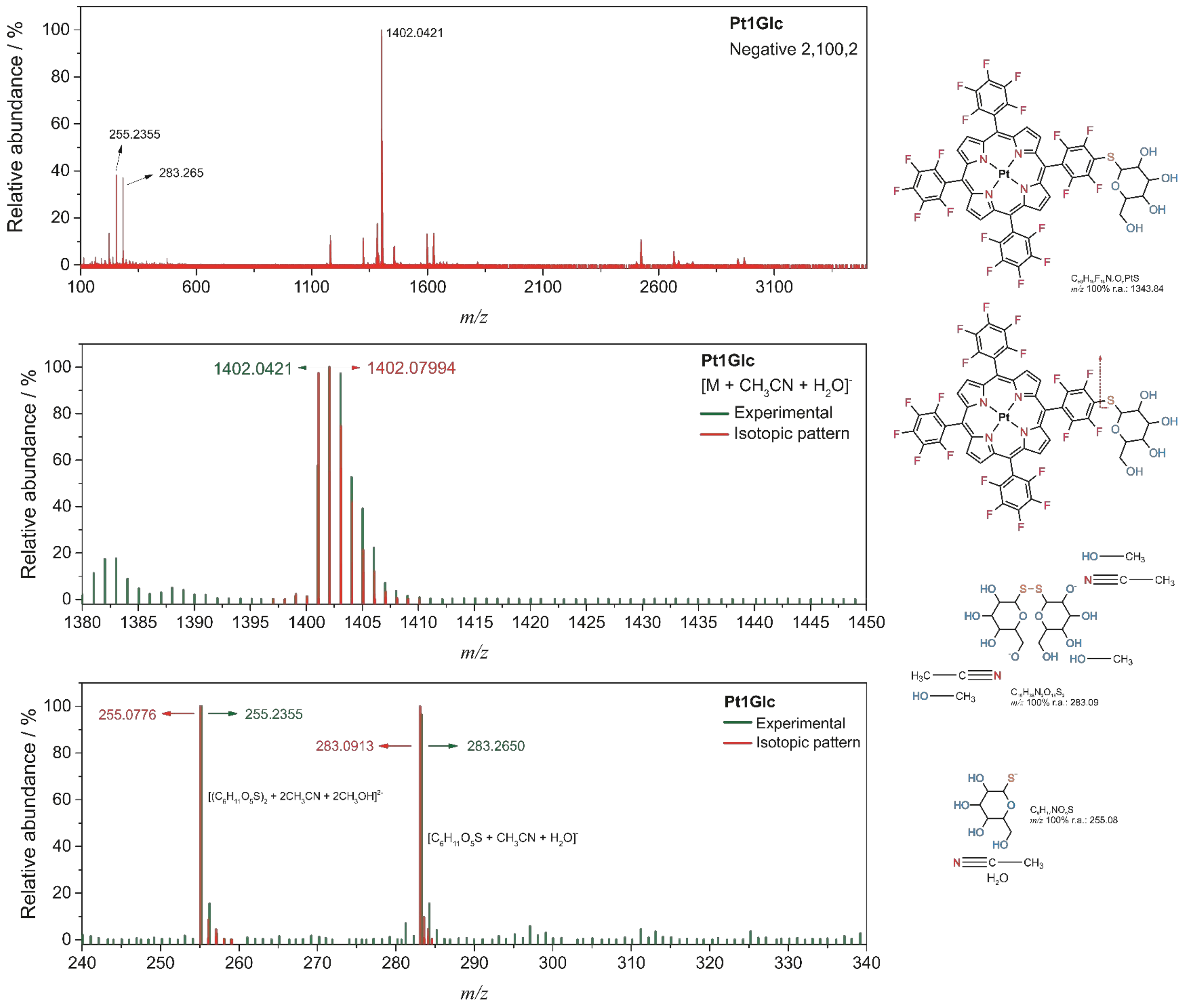

Spectra 20: HR-ESI Q-TOF mass spectra and isotopic pattern of the Pt1Glc compound.

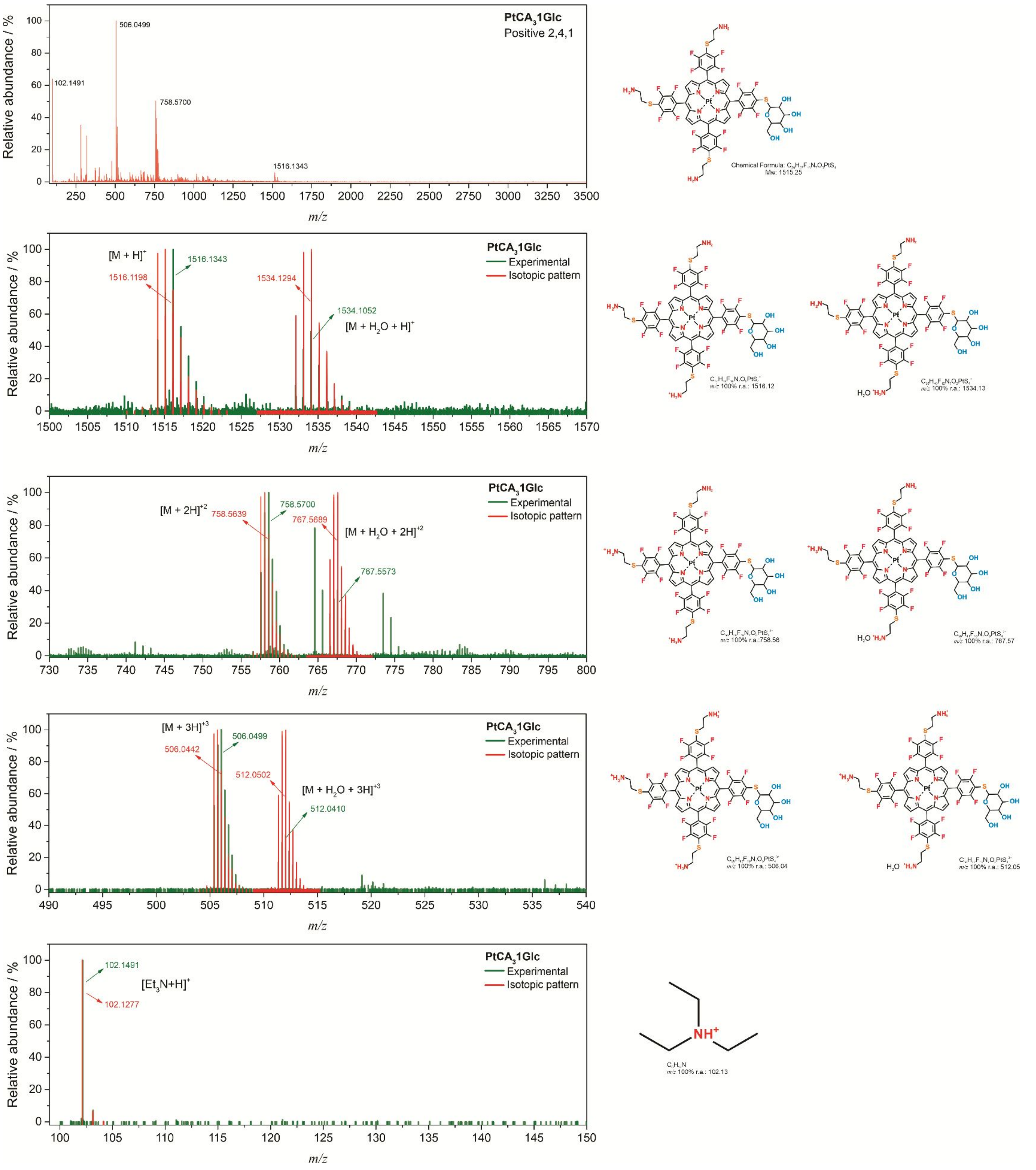

Spectra 21: HR-ESI Q-TOF mass spectra and isotopic pattern of the $PtCA_31Glc$ compound.

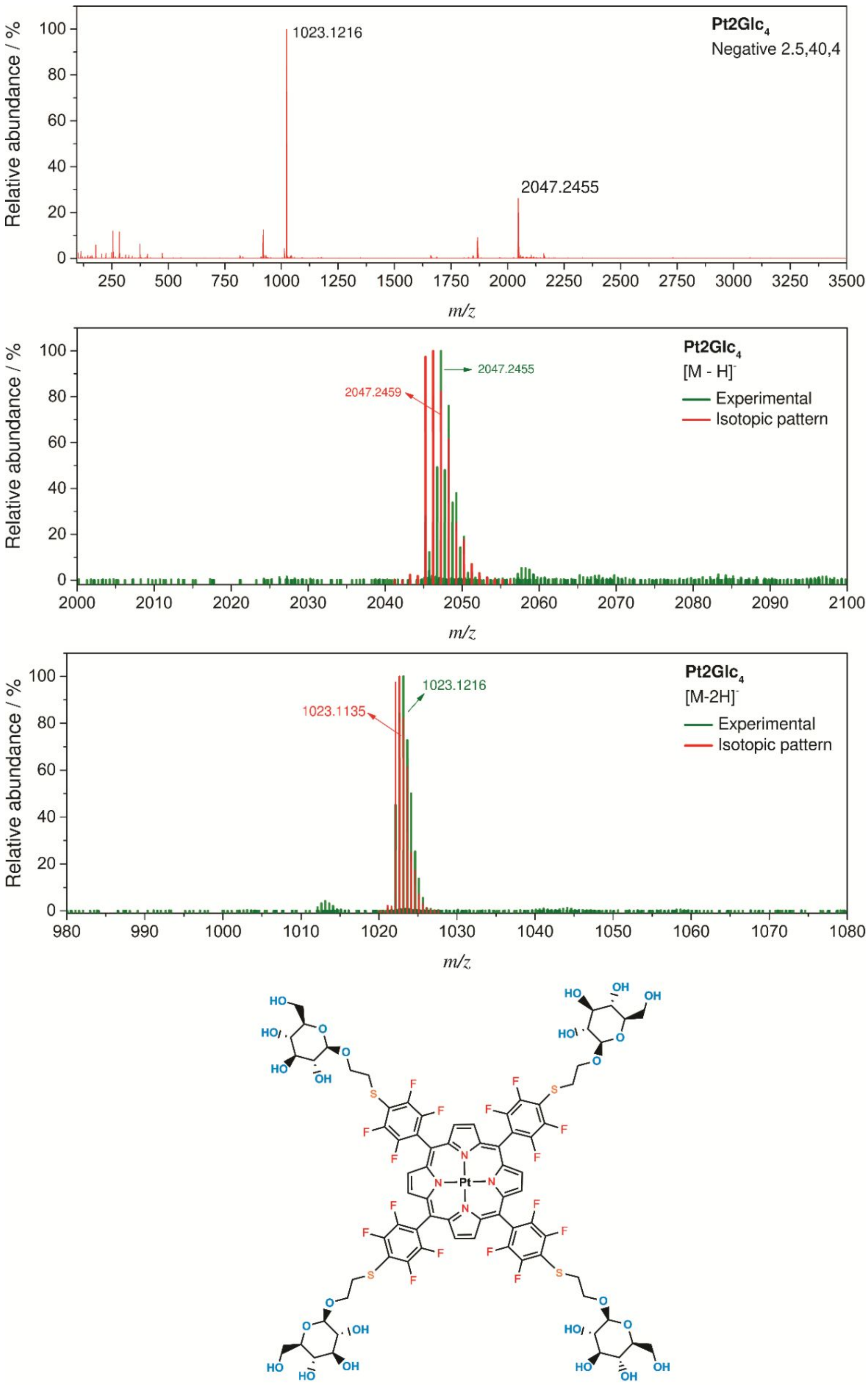

Chemical formula: $C_{76}H_{68}F_{16}N_4O_{24}PtS_4$
Mw: 2048.68

Spectra 22: HR-ESI Q-TOF mass spectra and isotopic pattern of the Ptc2Glc$_4$ compound.

4. Supplementary figures

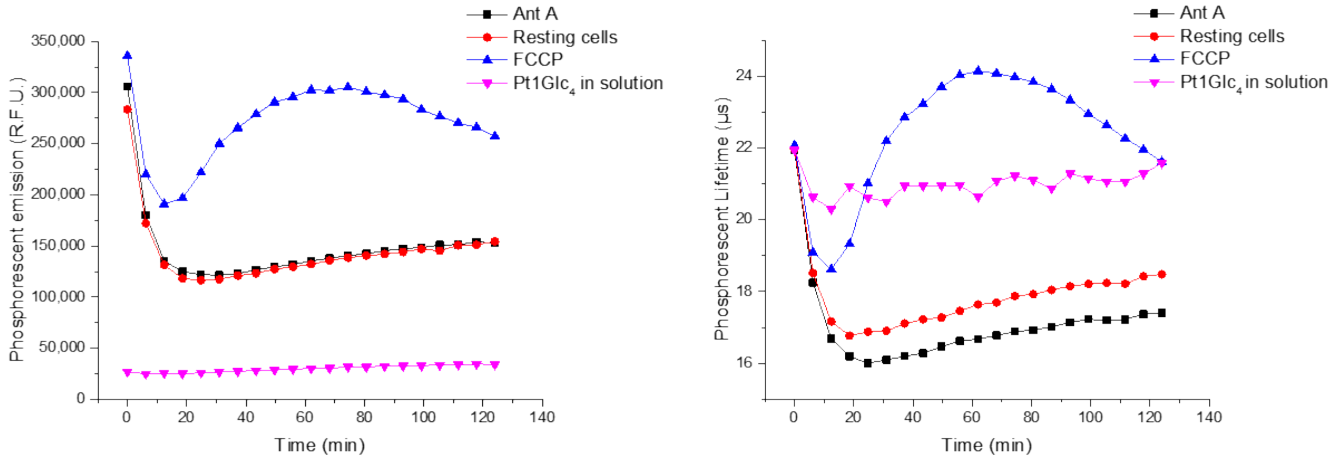

Figure S1: Phosphorescence intensity signals and calculated lifetimes produced by the intracellular probe $Pt1Glc_4$ (1µM), in glucose(+) medium and respiration profiles produced by the inhibition of mitochondrial complex III by Ant A (2 µM) or by FCCP uncoupling (0.25 µM).

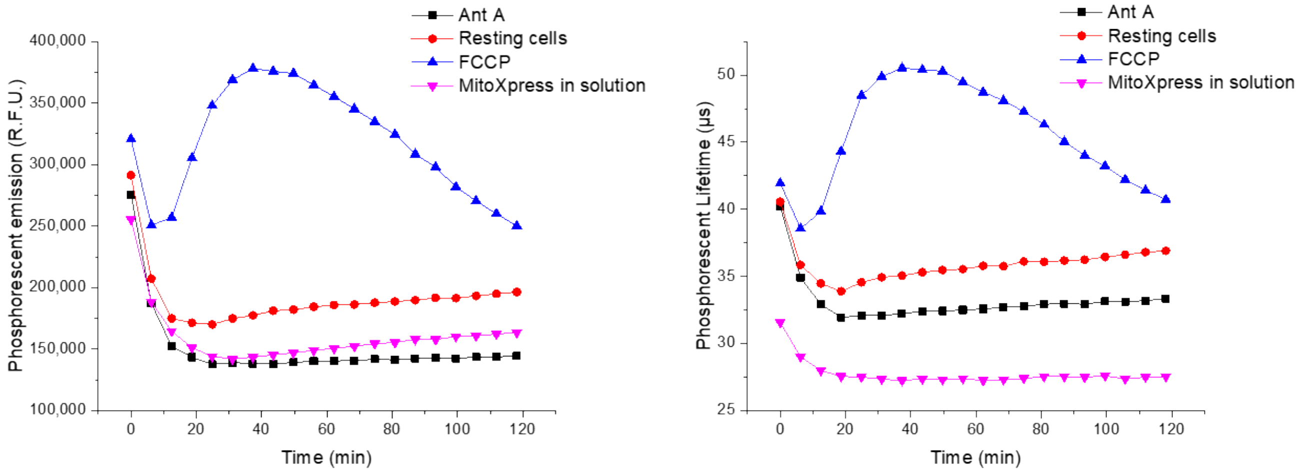

Figure S2: Phosphorescence intensity signals and calculated lifetimes produced by the extracellular probe MitoXpress®-Xtra in glucose(+) medium and respiration profiles produced by the inhibition of mitochondrial complex III by Ant A (2 µM) or by FCCP uncoupling (0.25 µM).

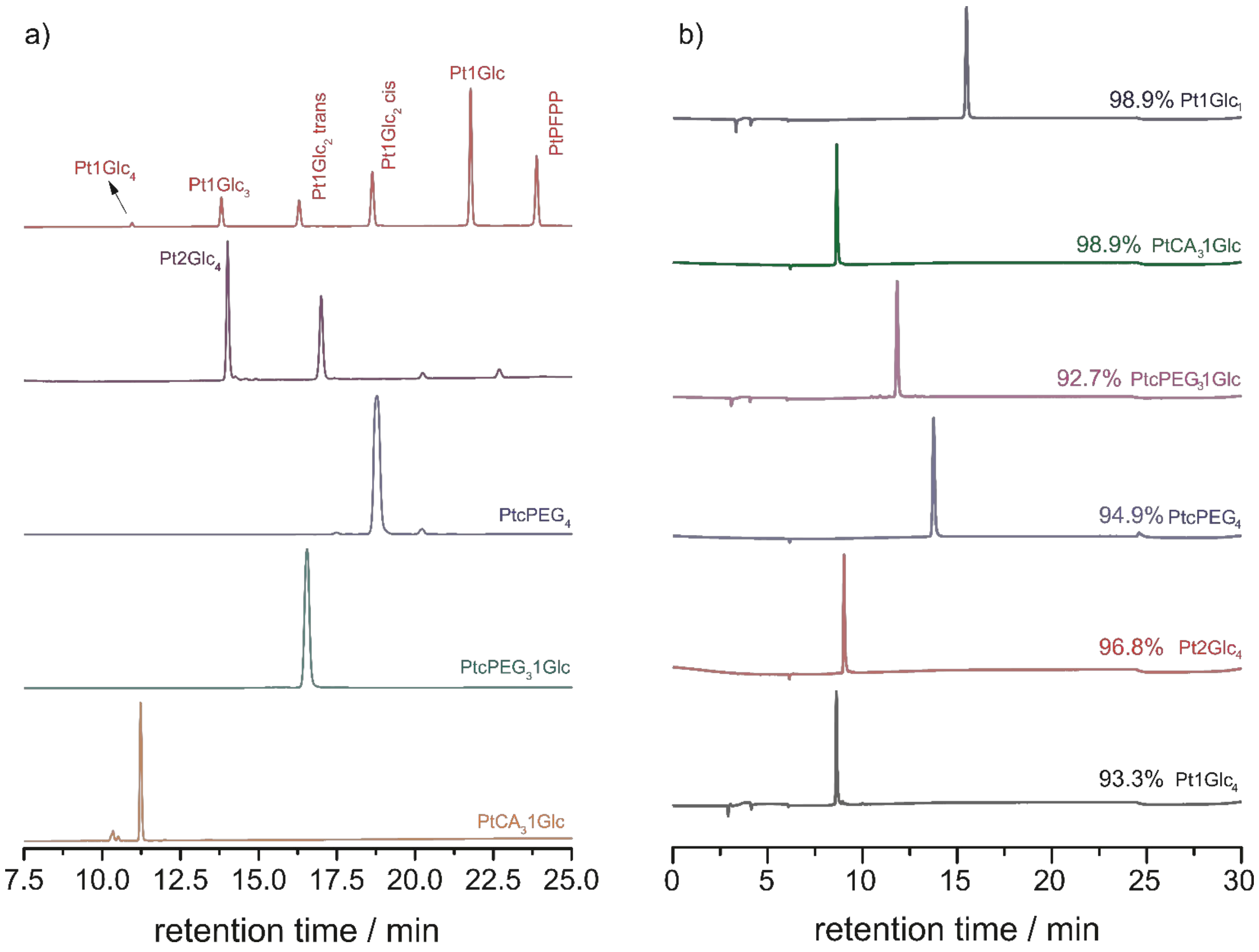

Figure S3: a) Analytical RP-HPLC (40 min gradient 0→100% of acetonitrile in aqueous 1% TFA and a flow rate of 0.63 ml/min) of the synthetic procedures and retention times of corresponding derivatives. b) HPLC traces for the obtained derivatives and their HPLC purity.

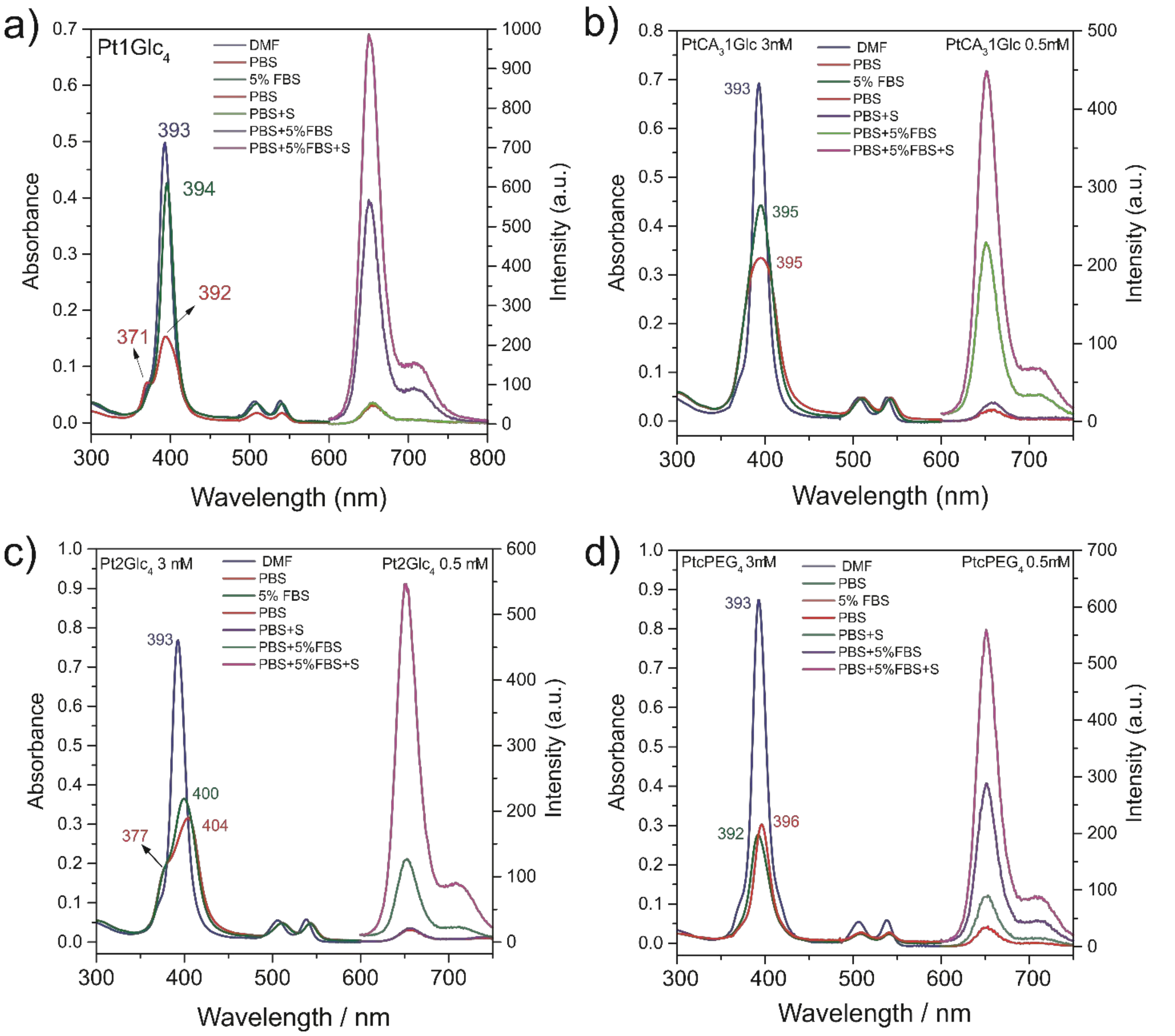

Figure S4: Absorbance and phosphorescence spectra of the produced derivatives in DMF, PBS, 5%FBS in oxygenated and deoxygenated conditions. A) Pt1Glc$_4$, b) PtCA$_3$1Glc, c) Pt2Glc$_4$, d) PtcPEG$_4$.
